# Cold-induced suspension and resetting of Ca^2+^ and transcriptional rhythms in the suprachiasmatic nucleus neurons

**DOI:** 10.1016/j.isci.2023.108390

**Published:** 2023-11-03

**Authors:** Ryosuke Enoki, Naohiro Kon, Kimiko Shimizu, Kenta Kobayashi, Sota Hiro, Ching-Pu Chang, Tatsuto Nakane, Hirokazu Ishii, Joe Sakamoto, Yoshifumi Yamaguchi, Tomomi Nemoto

**Affiliations:** 1 Biophotonics Research Group, Exploratory Research Center on Life and Living Systems (ExCELLS), National Institutes of Natural Sciences, Higashiyama 5-1, Myodaiji, Okazaki, Aichi 444-8787, Japan; 2 Division of Biophotonics, National Institute for Physiological Sciences, National Institutes of Natural Sciences, Higashiyama 5-1, Myodaiji, Okazaki, Aichi 444-8787, Japan; 3 School of Life Science, The Graduate University for Advanced Studies (SOKENDAI), Higashiyama 5-1, Myodaiji, Okazaki, Aichi 444-8787, Japan; 4 Institute of Transformative Bio-Molecules (ITbM), Nagoya University, Nagoya 464-8601, Japan; 5 Laboratory of Animal Integrative Physiology, Graduate School of Bioagricultural Sciences, Nagoya University, Nagoya 464-8601, Japan; 6 Suntory Rising Stars Encouragement Program in Life Sciences (SunRiSE), Suntory Foundation for Life Sciences, Kyoto 619-0284, Japan; 7 Department of Pathological Cell Biology, Medical Research Institute, Tokyo Medical and Dental University, Tokyo 113-8510, Japan; 8 Section of Viral Vector Development, National Institute for Physiological Sciences, Okazaki 444-8585, Japan; 9 Hibernation Metabolism, Physiology and Development Group, Institute of Low-Temperature Science, Hokkaido University, Sapporo, Hokkaido, Japan; 10 Graduate School of Environmental Science, Hokkaido University, Sapporo, Hokkaido, Japan; 11 Inamori Research Institute for Science Fellowship (InaRIS), Kyoto, Japan

**Keywords:** Biological sciences, Neuroscience, Molecular neuroscience, Cellular neuroscience

## Abstract

Does the circadian clock keep running under such hypothermic states as daily torpor and hibernation? This fundamental question has been a research subject for decades but has remained unsettled. We addressed this subject by monitoring the circadian rhythm of clock gene transcription and intracellular Ca^2+^ in the neurons of the suprachiasmatic nucleus (SCN), master circadian clock, *in vitro* under a cold environment. We discovered that the transcriptional and Ca^2+^ rhythms are maintained at 22°C–28°C, but suspended at 15°C, accompanied by a large Ca^2+^ increase. Rewarming instantly resets the Ca^2+^ rhythms, while transcriptional rhythms reach a stable phase after the transient state and recover their phase relationship with the Ca^2+^ rhythm. We conclude that SCN neurons remain functional under moderate hypothermia but stop ticking in deep hypothermia and that the rhythms reset after rewarming. These data also indicate that stable Ca^2+^ oscillation precedes clock gene transcriptional rhythms in SCN neurons.

## Highlights

• Transcriptional and Ca^2+^ rhythms are maintained at 22°C–28°C in SCN neurons
• Rhythms are suspended at 15°C, accompanied by a large Ca^2+^ increase
• Rewarming instantly resets the Ca^2+^ rhythm
• Stable Ca^2+^ rhythm precedes the transcriptional rhythms

## Graphical abstract

**Figure:**
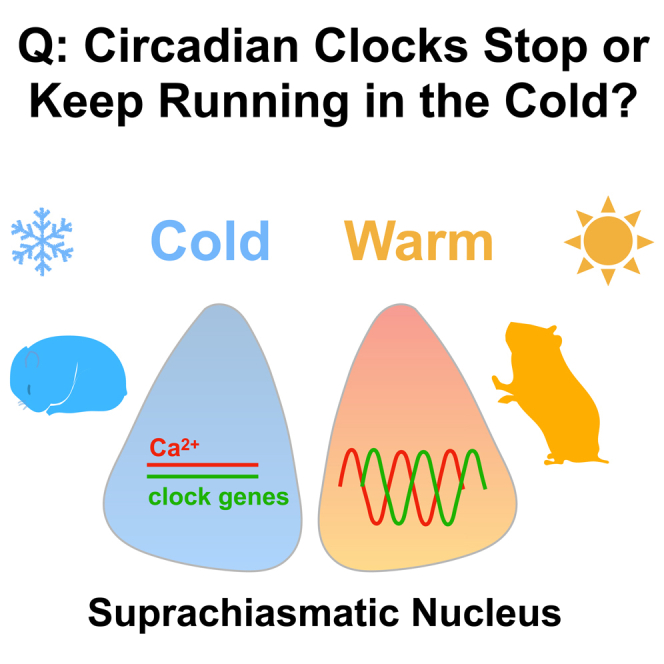

## Introduction

In mammals, daily rhythms in physiological and biochemical events, as well as animal behavior are coordinated by the master circadian clock localized in the hypothalamic suprachiasmatic nucleus (SCN) in the brain.^1^ In individual SCN neurons, cellular circadian rhythms are thought to be generated by an autoregulatory transcriptional and translational feedback loop (TTFL) comprising the core and sub-loop, composed of clock genes *Period (Per) 1*, *Per2*, *Cryptochrome (Cry) 1*, *Cry2*, *Bmal1*, and *Clock*, along with their protein products.^2^ The circadian clock has the unique property of exhibiting a temperature-compensated rhythm with a nearly 24-h periodicity. In general, a 10°C temperature increase accelerates the rate of biochemical reactions by a factor of 2–3 (Q_10_ = 2 to 3), while the Q_10_ of the circadian period is compensated to 0.8 to 1.2. This phenomenon is referred to as “temperature compensation,” an essential intrinsic circadian clock property.

In the core transcriptional feedback loop, CLOCK and BMAL1 activate the transcription of *Per1*/*2* and *Cry1/2* through E-box *cis*-element, and resultant PERs and CRYs interfere with transcriptional activation by CLOCK-BMAL1 heterodimers.^1^^,^^3^ In addition, *Per1*/2 are regulated by Ca^2+^/cAMP response element binding protein (CREB) through CRE. Because transcriptional activities of CLOCK and CREB are regulated by Ca^2+^/calmodulin-dependent protein kinase II (CaMKII), expression levels of *Per1/2* are directly regulated by intracellular Ca^2+^ dynamics.^1^^,^^3^ *Bmal1* is rhythmically transcribed through ROR/REV-ERB responding element (RRE), and this transcriptional regulation forms the sub-loop.^2^ *Per2* and *Bmal1* are reported to regulate Ca^2+^ oscillations,^4^^,^^5^ indicating that TTFL couples with Ca^2+^ oscillation in the SCN neurons. Moreover, it has been suggested that Ca^2+^ oscillation acts as a cytosolic messenger linking TTFL and physiological activities in the SCN neurons.^6^^,^^7^ Importantly, we previously demonstrated that the Ca^2+^ oscillation sustains even in the SCN neurons of *Cry1/2* deficient mice, in which the TTFL is disrupted.^8^ In addition, our recent study revealed a conserved role of cold-responsive Ca^2+^ signaling for temperature-compensated oscillations of TTFL.^9^ These results lead to the idea that the Ca^2+^ signaling is a primary timekeeping mechanism to sustain TTFL oscillations.

Various mammalian species use evolutionarily developed controllable hypothermia, torpor, and hibernation, by lowering thermogenesis and basal metabolism to survive during the harsh winter season for food.^10^^,^^11^^,^^12^ Mammalian hibernators, such as squirrels and hamsters, undergo deep hypothermia, during which their core body temperature reaches a comparable level of ambient temperature. This deep hypothermic state is maintained for several hours or days, or over a week in some species, interrupted by rewarming-related arousal through nonshivering and shivering thermogenesis. Mice enter a relatively shallow form of hypothermia, daily torpor that reduces their energy expenditure to survive when food is unavailable at any time of the year.^13^ During this moderate hypothermia, the core body temperature drops to around 20°C–25°C and rises back to the normothermic state after several hours.

The entry of a hypothermic state, in both torpor and hibernation, is reportedly time-dependent with a circadian clock-regulated timing,^12^^,^^14^^,^^15^ although several counterarguments exist as well.^16^ In addition, the physiological rhythms display arrhythmia after post-hibernation,^17^ suggesting that the time-keeping system is affected by rewarming from low temperatures. Multiple studies have addressed the fundamental question of whether circadian rhythms continue to function during hypothermia, although in general, most reports indicate that it does not with body temperatures around 10°C.^18^^,^^19^^,^^20^^,^^21^ However, several studies have reported the presence of a functional circadian rhythm at body temperatures above 10°C.^22^^,^^23^ Nevertheless, as these studies were conducted in different animal species and experimental environments, continuous analysis of circadian rhythms in identical tissues and in a temperature-controlled environment is warranted.

Pioneering work established time-lapse fluorescence imaging of intracellular Ca^2+^ at the single-cell level in SCN slices,^24^ and further studies extended this approach to network and multicolor imaging, reporting the underlying cellular and network mechanisms of circadian Ca^2+^ rhythms in the SCN.^8^^,^^25^^,^^26^^,^^27^^,^^28^ In this study, we further extended these approaches to optical imaging under a controllable temperature environment and performed simultaneous dual-color fluorescence imaging of the clock genes and intracellular Ca^2+^ rhythms in SCN slices. We observed that the clock gene and Ca^2+^ circadian rhythms are maintained in the cold at 22°C–28°C, but suspended at approximately 15°C. Rewarming immediately resets the circadian Ca^2+^ rhythms, whereas the *Bmal1* and *Per2* rhythms gradually reestablish the phase relation relative to the Ca^2+^ rhythm. Our data demonstrate that stable Ca^2+^ oscillation precedes clock gene transcriptional rhythms in the SCN neurons.

## Results

### Low-temperature time-lapse fluorescence imaging in SCN neurons

**Figure 1:**
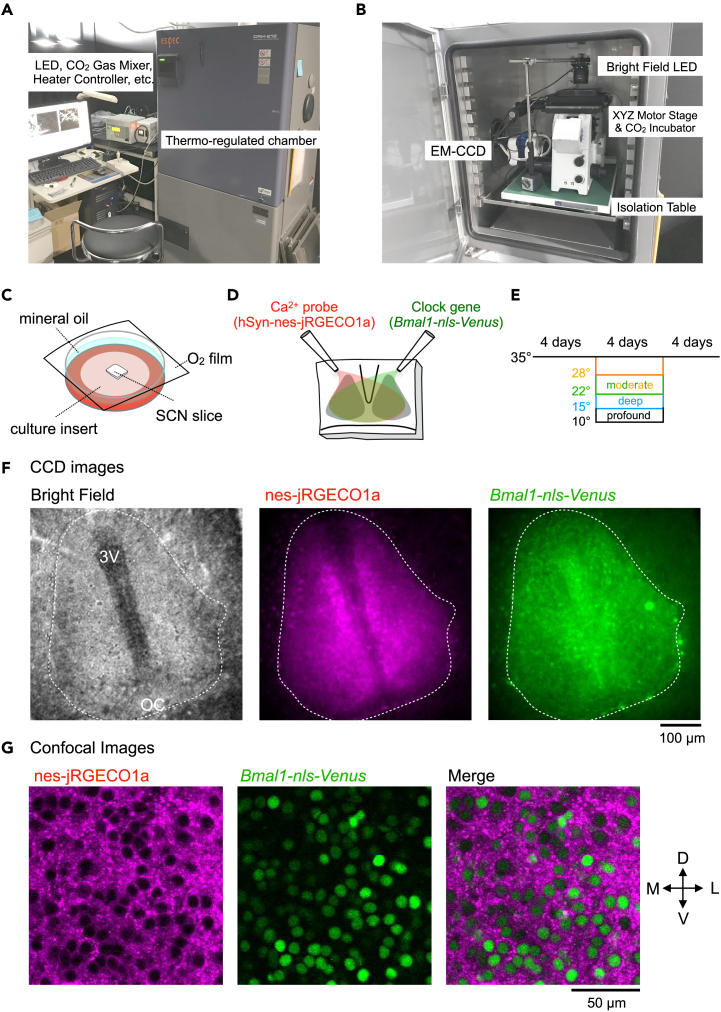
Low-temperature time-lapse fluorescence imaging in mouse SCN slices (A) External view of the low-temperature imaging system. (B) Interior view of the microscopic platform in the thermo-regulated chamber. (C) Slice culture in the glass-bottom dish. The surface of the culture medium is covered with mineral oil, and the culture dish is sealed with an O_2_-permeable filter. (D) adeno-associated virus (AAV) infection in SCN slices. An aliquot of the AAV harboring Syn1-nes-jRGECO1a (nes-jRGECO1a) and AAV-Bmal-forward-intron336-Venus-NLS-D2 (*Bmal1-nls-Venus*) are inoculated onto the surface of the SCN slices. (E) Schedule for time-lapse imaging. After 4 days recording of the Ca^2+^ and *Bmal1* rhythms at 35°C, the SCN slices are chilled at various low temperatures for 4 days, followed by rewarming to 35°C for 4 days. In this study, we defined 15°C, 22°C and 28°C, and 10°C as deep, moderate, and profound hypothermia, respectively. (F) Representative images of mouse SCN slices expressing nes-jRGECO1a and *Bmal1-nls-Venus*. Bright-field (left), nes-jRGECO1a (center), *Bmal1-nls-Venus* (right). (G) Confocal images of nes-jRGECO1a (left), *Bmal1-nls-Venus* (center), and merged images on the left side of the SCN slices. The center region of the left SCN is depicted. 3V: the third ventricle, OC: optic chiasma.

To continuously monitor the circadian rhythms in the SCN neurons in a temperature-controlled environment, we constructed a time-lapse fluorescence imaging system composed of an epifluorescence microscope placed inside a thermo-regulated chamber (Figures 1A and 1B). The surface of the culture medium was covered with noncytotoxic mineral oil, and the culture dish was sealed with an O_2_-permeable filter, enabling us to monitor the SCN slices without temperature difference-related water condensation and evaporation during the long day recordings (Figure 1C). We used the yellow fluorescence protein reporter Venus under the control of a *Bmal1* promoter (Bmal1-forward-intron336-Venus-NLS-D2; *Bmal1-nls-Venus*), and a red-color Ca^2+^ probe under a neuron-specific promoter (Syn1-nes-jRGECO1; nes-jRGECO1a) for visualization. We prepared mouse SCN slices and expressed these fluorescence probes by the adeno-associated virus (AAV) (Figure 1D). To quantitatively evaluate the periodicity and continuity of the circadian rhythm, unless otherwise stated, the recording period was set for 4 days under various temperature conditions, and the total recording time was set for 12 days. We chilled the SCN slices under low temperatures, followed by rewarming to 35°C (Figure 1E). Based on the previously reported classification of hypothermia,^29^ we defined 15°C, 22°C–28°C, and 10°C as deep, moderate, and profound hypothermia, respectively, in this study. Figure 1F presents the typical expression patterns of nes-jRGECO1a and *Bmal1-nls-Venus* in the SCN slices. The high-resolution confocal images of the SCN slices (Figure 1G), together with our previous studies on the Ca^2+^ probe expression patterns,^25^^,^^26^ indicate that nes-jRGECO1a and *Bmal1-nls-Venus* are exclusively expressed in the SCN neuronal cytoplasm and nucleus, respectively.

### Ca^2+^ and *Bmal1* rhythms under cold temperatures

**Figure 2:**
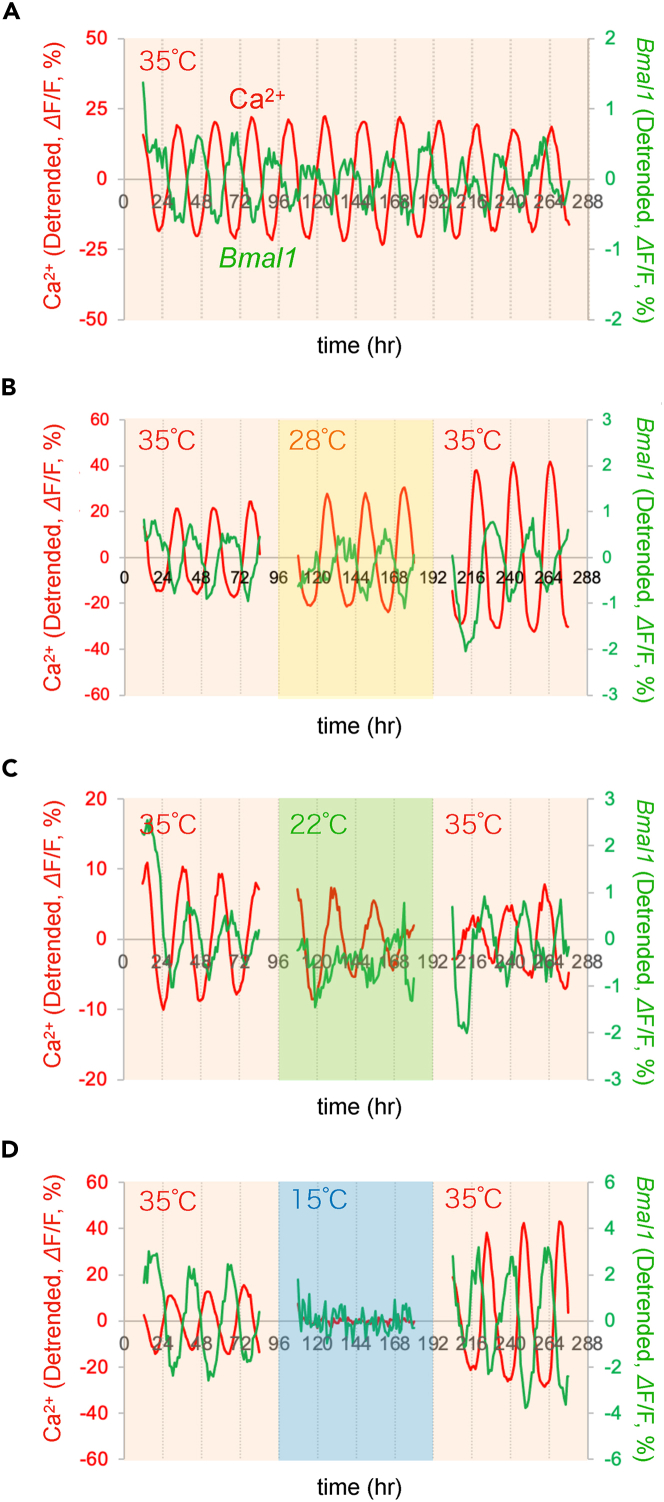
Ca^2+^ and *Bmal1* rhythms at various temperatures Representative traces of Ca^2+^ and *Bmal1* rhythms. (A–D) After recording Ca^2+^ and *Bmal1* rhythms for 4 days at 35°C, mouse SCN slices were exposed to various temperatures for 4 days (top to bottom panels: 35°C, 28°C, 22°C, and 15°C), followed by rewarming to 35°C. All traces represent the mean signals in the whole SCN region, and the traces were detrended by a 24-h running average subtraction method. Time is depicted after the start of the recording.

**Figure 3:**
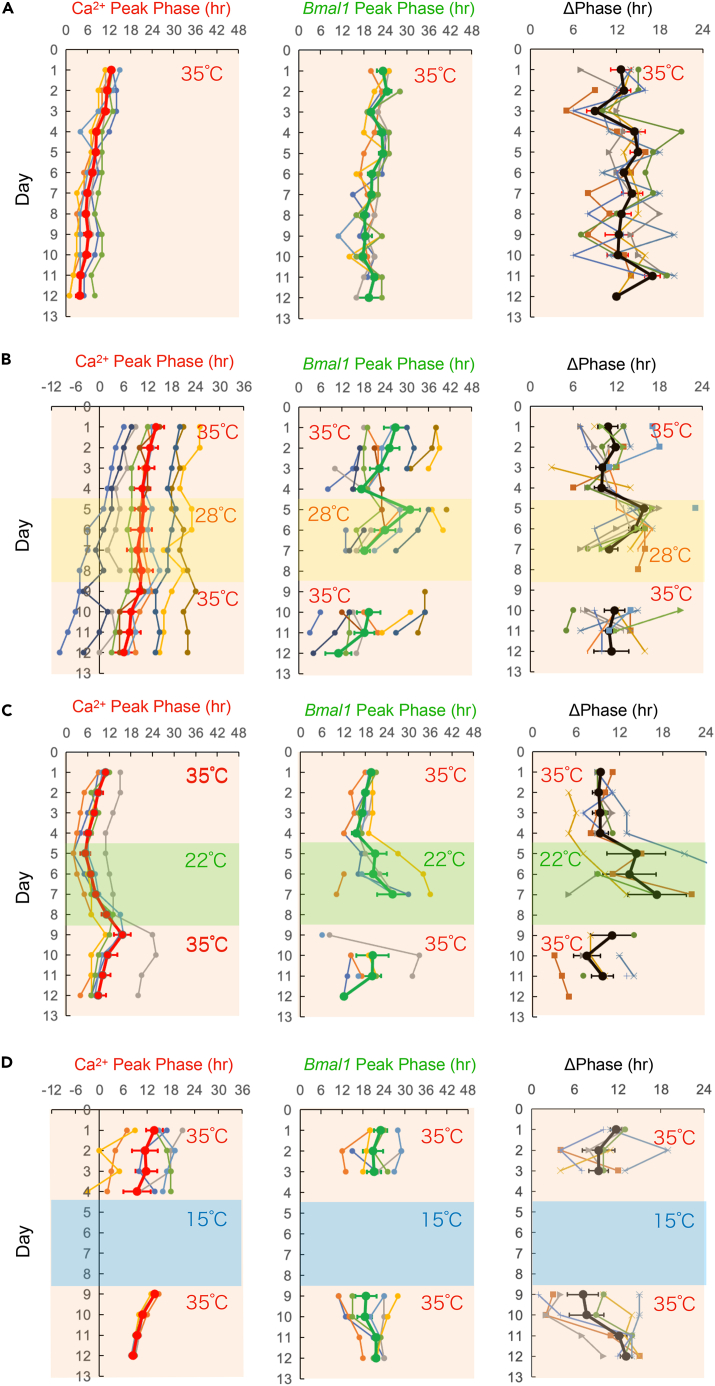
The plots of Ca^2+^ and *Bmal1* rhythms and phase difference in SCN neurons (A–D) Ca^2+^ and *Bmal1* rhythms were recorded at 35°C for 4 days, and then the SCN slices were exposed to various temperatures for 4 days, followed by rewarming to 35°C for another 4 days. Ca^2+^ rhythm (left), *Bmal1* rhythm (center), and the phase difference (right). Time is depicted after the start of the recording. Individual slice data and average data are shown. The mean data are presented as the mean ± SEM.

**Figure 4:**
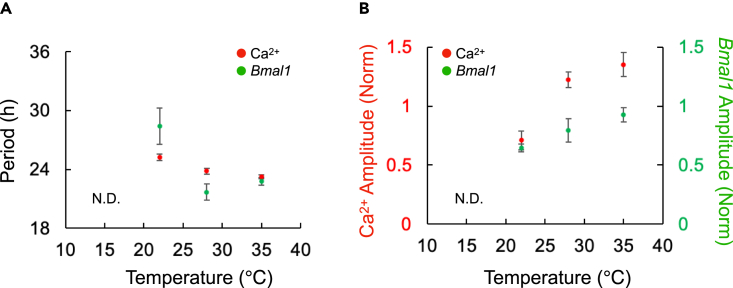
Summary of Ca^2+^ and *Bmal1* periods and amplitudes at various temperatures (A and B) Graphs represent the relationship between period (A) or amplitude (B) vs. various temperatures (35°C, 28°C, and 22°C). Rhythms were not detectable at 15°C (N.D.). The amplitudes were normalized (Norm) by values before temperature change. The data are presented as the mean ± SEM.

**Figure 5:**
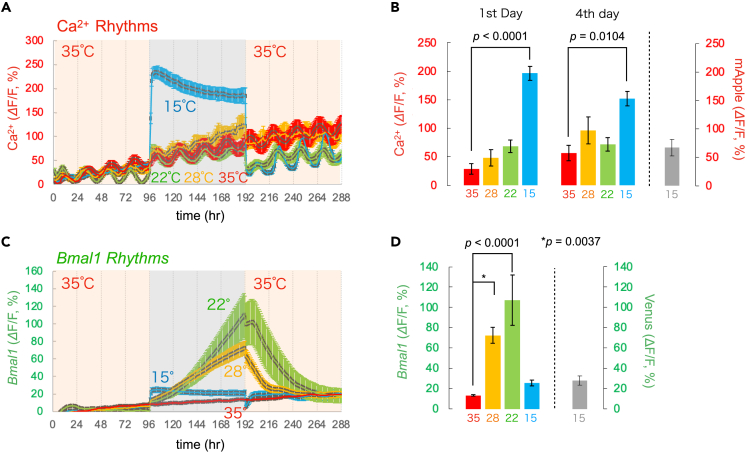
Baseline levels of Ca^2+^ and *Bmal1* signals at various temperatures in SCN neurons (A) Baseline Ca^2+^ levels at 35°C, 28°C, 22°C, and 15°C. (B) Statistical comparison of baseline Ca^2+^ levels on Days 1 and 4 under cold temperature conditions. Right gray bar: mApple fluorescence at 15°C. (C) Baseline levels of *Bmal1* signal at 35°C, 28°C, 22°C, and 15°C. (D) Statistical comparison of the *Bmal1* signal maximum baseline levels under cold temperature conditions. Right gray bar: Venus fluorescence alone at 15°C. Time is depicted after the start of the recording. All data are presented as the mean ± SEM.

We monitored the Ca^2+^ and *Bmal1* signals in the SCN slices at various temperatures (35°C, 28°C, 22°C, 15°C, and 10°C) and assembled on figures the representative traces (Figure 2), the peak phase plots of the rhythms (Figure 3), the statistical comparison of the period and amplitude (Figure 4), and the baseline level (Figure 5). In most of the figures, we analyzed the rhythms in the whole SCN regions. In Figure S8, we performed single-cell analysis.

First, to assess the stability and continuity of the circadian rhythms under our experimental conditions, we measured Ca^2+^ and *Bmal1* signals in the SCN slices for 12 days at 35°C (Figure 2A). We detected stable Ca^2+^ and *Bmal1* rhythms with periods slightly shorter than 24 h (Ca^2+^: 23.2 ± 0.1 h, *Bmal1*: 23.6 ± 0.1 h) and a nearly anti-phasic relation (13.2 ± 0.6 h) (n = 6 slices), the value of which was consistent with the results of a previous study.^30^

The SCN slices were then exposed to a cold temperature of 28°C for 4 days, followed by rewarming to 35°C for another 4 days (Figure 2B). Such low-temperature mimics moderate hypothermia present during fasting-induced torpor in mice^13^ and induced hypothermia by the activation of a discrete subset of neurons in the hypothalamus.^31^^,^^32^ Multiple circadian peaks could be detected in this cold (Figure 2B), indicating the persistent rhythmicity of both Ca^2+^ and *Bmal1* (Figure 2B). After rewarming to 35°C, both the Ca^2+^ and *Bmal1* rhythms were maintained with an anti-phase relationship. We then further chilled the SCN slices at 22°C. We observed that the Ca^2+^ and *Bmal1* rhythms persisted even under such cold conditions (Figure 2C). After rewarming to 35°C, both the Ca^2+^ and *Bmal1* rhythms were maintained with an anti-phase relationship.

Next, the SCN slices were chilled at 15°C, mimicking deep hypothermia (Figure 2D). Notably, neither Ca^2+^ nor *Bmal1* rhythms could be detected at this cold temperature (Figure 2D right). After rewarming to 35°C, both the Ca^2+^ and *Bmal1* rhythms appeared and continued. Taken together, these results indicate that the circadian rhythms suspend at temperatures as cold as 15°C and that the critical point for the circadian rhythm suspension is around 15°C–22°C.

Under physiological conditions in mice, the core body temperature is not expected to drop below 15°C–22°C, although it does indeed during controlled anesthesia.^33^^,^^34^ We did not detect signs of tissue damage at 15°C–35°C (Figures S1A–S1D). However, at 10°C, especially after rewarming to 35°C, we detected severe tissue damage and shrinkage (Figure S1E). These results indicate that the SCN in mice cannot tolerate exposure to cold temperatures below 10°C, we thus excluded these data from further analysis.

We further evaluated the condition of SCN neurons using a dying cell maker (SYTOX-Green) and performed time-lapse imaging under cold exposure (Figure S2). We added ionomycin and CaCl_2_ to maximize intracellular Ca^2+^ concentration and cellular damage, leading to the strongest SYTOX-Green signal (ΔF/F_0_, 600%) (Figure S2A). Next, we performed a simultaneous time-lapse imaging of SYTOX-Green and nes-jRGECO1a (Figures S2C–S2F). At 35°C, Ca^2+^ rhythms were observed for 4 days in the SCN slices and a weak SYTOX-Green signal was detected in a small subset of cells. This result indicates that SYTOX-Green did not perturb the Ca^2+^ rhythm generations. At 15°C, we detected a few SYTOX-Green signal spots, especially after 3–4 days of cold exposure. The signal intensity was approximately 60% (ΔF/F_0_) and the number of spots was considerably lower than that obtained using ionomycin and CaCl_2_. After rewarming, the signal spots quickly returned to undetectable levels. We observed small tissue shrinkage after rewarming, probably due to the elimination of damaged cells after rewarming. Based on these observations, we concluded that a long cold exposure at 15°C for 3–4 days causes mild cell damage in a small population of cells; however, damage can be restored and the cells can be recovered after rewarming.

### Statistical evaluation of Ca^2+^ and *Bmal1* rhythms under cold temperatures

At a constant temperature of 35°C, the Ca^2+^ and *Bmal1* rhythms with an anti-phasic relationship could be detected for 12 days in the SCN neurons (Figure 3A) (n = 6 slices). The Ca^2+^ and *Bmal1* rhythm periods were shorter than 24 h, remaining stable during the long recording for 12 days (Ca^2+^ rhythm: 22.6 ± 0.5, 23.3 ± 0.2, and 23.6 ± 0.1 h on Days 1–4, 5–8, and 9–12, respectively; *Bmal1* rhythm: 23.3 ± 0.5, 22.8 ± 0.4, and 24.8 ± 0.6 h on Days 1–4, 5–8, and 9–12, respectively) (Figure S3A1). The Ca^2+^ and *Bmal1* rhythm amplitudes were also stable over the long recording (Ca^2+^ rhythm: 23.3 ± 4.7%, 29.8 ± 4.8%, and 29.9 ± 5.2% on Days 1–4, 5–8, and 9–12, respectively; *Bmal1* rhythm: 1.5 ± 0.3%, 1.4 ± 0.3%, and 1.5% ± 0.4% on Days 1–4, 5–8, and 9–12, respectively) (Figure S3A2).

Under cold temperatures of 28°C, the Ca^2+^ rhythm phase remained stable, whereas the *Bmal1* rhythm was transiently delayed (Figure 3B, center), and the phase difference between the Ca^2+^ and *Bmal1* rhythms widened (Day 5 vs. Day 4, p = 0.0482). After this transient delay, the *Bmal1* rhythm phase advanced and the phase difference returned to the pre-chilling level on Day 7. At 28°C, the Ca^2+^ rhythm period slightly lengthened (Figure S3B1) (22.9 ± 0.1, 23.9 ± 0.3, and 22.8 ± 0.2 h at 35°C, 28°C, and after rewarming, respectively; p = 0.006) (n = 10 slices), whereas the *Bmal1* rhythm did not (23.9 ± 1.1, 21.7 ± 0.8, and 22.9 ± 1.3 h at 35°C, 28°C, and after rewarming, respectively). Our statistical analysis showed that the *Bmal1* rhythm amplitudes became significantly smaller (1.5 ± 0.2%, 1.1 ± 0.1%, and 1.7 ± 0.2% at 35°C, 28°C, and after rewarming, respectively; p = 0.034), although it was not the case for Ca^2+^ rhythms (31.9 ± 3.8%, 39.9 ± 5.7%, and 43.1 ± 9.5% at 35°C, 28°C, and after rewarming, respectively) (Figure S3B2). These results show that both Ca^2+^ and *Bmal1* circadian rhythms persist at moderate cold temperatures of 28°C and that the Ca^2+^ rhythm remains stable, but the *Bmal1* rhythm is more sensitive to temperature changes.

At the cold temperature of 22°C, the peak phases of the Ca^2+^ and *Bmal1* rhythms were delayed (Figure 3C, left and center) (n = 6 slices) and the Ca^2+^ and *Bmal1* rhythm phase differences gradually widened during this cold exposure (Figure 3C, right). The periods of the two rhythms significantly lengthened (Ca^2+^ rhythm: 22.4 ± 0.1, 25.3 ± 0.3, and 22.1 ± 0.3 h at 35°C, 28°C, and after rewarming, respectively, p = 0.0006; *Bmal1* rhythm: 22.5 ± 0.4, 28.4 ± 1.8, and 24.9 ± 1.0 h at 35°C, 28°C, and after rewarming, respectively, p = 0.0342) (Figure S3C1). Notably, the *Bmal1* period was significantly larger in Ca^2+^ rhythms (p = 0.0482). After rewarming, the period was returned to that of the pre-chilling levels (Figure S3C1). The Ca^2+^ and *Bmal1* rhythm amplitudes were significantly attenuated (Ca^2+^ rhythm: 34.9 ± 5.3%, 25.2 ± 5.3%, and 26.6 ± 6.8% at 35°C, 28°C, and after rewarming, respectively, p = 0.0388; *Bmal1* rhythm: 2.0 ± 0.2%, 1.3 ± 0.2%, and 2.0 ± 0.2% at 35°C, 28°C, and rewarming, respectively, p = 0.0006) (Figure S3C2). These results showed that both the Ca^2+^ and *Bmal1* rhythms persist at the temperature of 22°C and these two rhythms transiently dissociate under such cold conditions.

Upon exposing the cells to low temperature at 15°C, no Ca^2+^ and *Bmal1* rhythms were detected (Figure 3D). After rewarming, both Ca^2+^ and *Bmal1* rhythms reappeared but their peak phases showed distinct patterns; the peak phase of Ca^2+^ rhythm was detected at 14.0 ± 0.3 h (n = 6), whereas that of *Bmal1* was widely distributed on Day 1 after rewarming (SD = 7.9 h). Both rhythms reached a stable phase after a longer transient state (Figure 3D, left and center).

Figure 4 summarizes the temperature-dependent changes in the Ca^2+^ and *Bmal1* rhythm periods and amplitudes. The Ca^2+^ rhythm periods were relatively constant compared to those of the *Bmal1* rhythms (Figure 4A). The amplitude of both rhythms decreased monotonically (Figure 4B). The critical temperature for the rhythm suspension was at approximately 15°C and below 22°C, respectively.

### Cold exposure increases the Ca^2+^ and *Bmal1* baseline levels

Cold exposure at 22°C–28°C increased the Ca^2+^ rhythm baseline levels (Figure 5A). Notably, gradual fluorescence intensity increases could be detected during the long recordings, typically occurring in AAV-transfected SCN neurons.^8^^,^^25^^,^^35^ Figure 5B presents the Ca^2+^ baseline level comparisons on Days 1 and 4 (Day 1: 28.7 ± 9.2%, 48.2 ± 14.7%, and 68.4 ± 11.0% at 35°C, 28°C, and 22°C, respectively; Day 4: 56.7 ± 13.3%, 96.6% ± 23.6%, and 71.9 ± 11.7% at 35°C, 28°C, and 22°C, respectively). At 15°C, the Ca^2+^ baseline level increased rapidly and reached significantly higher levels on Day 1 (196.7 ± 12.3%, p < 0.0001) and Day 4 (152.2 ± 12.6%, p = 0.0104). After rewarming to 35°C, the baseline levels returned quickly to lower values. In contrast, the *Bmal1* baseline level gradually increased during the 22°C–28°C exposure (13.4 ± 0.8%, 72.5 ± 7.8%, and 107.1 ± 24.7% at 35°C, 28°C, and 22°C, respectively, p = 0.0037 for 28°C, p < 0.0001 for 22°C) (Figures 5C and 5D). In the case of 15°C cold exposure, the increase in the baseline level of the *Bmal1* rhythm was smaller and flattened (25.4 ± 2.9%).

To further confirm whether nes-jRGECO1a could detect an increase in Ca^2+^ during cold exposure, we increased the intracellular Ca^2+^ concentration to the maximum level by supplementing the medium with 10 μM ionomycin and 20 mM CaCl_2_ at 15°C. This treatment further increased nes-jRGECO1a signal during cold exposure at 15°C (Figure S4), indicating that the upregulated Ca^2+^ level under cold exposure at 15°C was within the detectable range of nes-jRGECO1a.

To understand the biophysical properties of nes-jRGECO1a at various temperatures, we purified the nes-jRGECO1a protein and measured its fluorescence spectrum using a spectrometer at various temperatures (5°C–35°C, with 5°C increments) (Figure S5). Fluorescent proteins are known to exhibit brighter signals with decreasing temperatures,^36^ and the same was observed in the case of purified nes-jRGECO1a (Figure S5A). We confirmed that the KD and Hill coefficient of nes-jRGECO1a did not change abruptly at a certain temperature (Figure S5B). These results indicate that the biophysical properties of the probe cannot explain the upregulation of Ca^2+^ level under cold exposure at 15°C in the SCN neurons.

Next, we investigated the temperature dependence of the fluorescent protein signals in living SCN cells. Venus and mApple proteins (the base protein of jRGECO1a) were virally expressed in the SCN slices. As expected, we observed increased Venus and mApple fluorescent signals upon cold exposure at 15°C (27.7 ± 4.6%, n = 3, 67.1 ± 14.6%, n = 3, respectively) (Figure S6). *Bmal1* baseline levels at 28°C, but not at 22°C and 15°C, were significantly higher than Venus intensity (p = 0.0115 for 28°C, unpaired t test). The Ca^2+^ baseline levels on Day1 and Day4 at 15°C, but not at 22°C–28°C, were significantly higher than mApple intensity (p = 0.0004 for Day 1, p = 0.0046 for Day 4, respectively, unpaired t test) (Figures 5B and 5D). From these observations, we concluded that Ca^2+^ rhythms, unlike *Bmal1* rhythms, significantly increase under cold exposure at 15°C.

Fluorescent probes often suffer from pH sensitivity. To confirm the findings of Ca^2+^ signal increase and rhythm suspension at 15°C, and rhythm resetting after rewarming, we performed time-lapse imaging using an FRET Ca^2+^ probe, Twitch2B, a troponin C-based Ca^2+^ probe.^37^ We observed circadian signal changes of mCerulean and cpVenus, an FRET pair of Twitch2B, and signals changed in the opposite intensity direction at 35°C and 15°C (Figure S7). This result further supports our conclusion related to the Ca^2+^ signals under cold exposure.

### Rewarming differentially restarts the *Bmal1* and *Per2* circadian rhythms

Next, we compared the cold responses of two transcriptional rhythms, *Bmal1* and *Per2*. The *Bmal1* transcription depends on RORs and REVERBs present in the upstream region of *Bmal1*, whereas *Per2* transcription is regulated through the cAMP responding element (CRE) or E-box by CREB or CLOCK.^1^ Because CREB and CLOCK are regulated by Ca^2+^-CaMKII (32), *Per2* is directly regulated by Ca^2+^ signaling.

**Figure 6:**
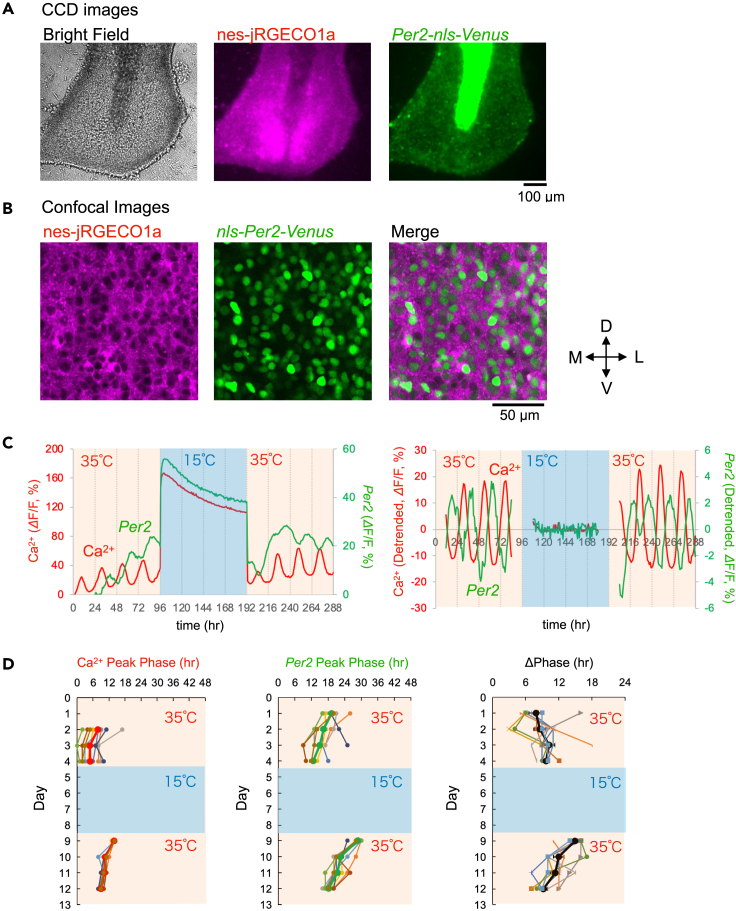
Ca^2+^ and *Per2* rhythms exposed to 15°C (A) Representative images of mouse SCN slices expressing nes-jRGECO1a and *Per2-nls-Venus*. Brightfield (left), nes-jRGECO1a (center), and *Per2-nls-Venus* (right). (B) Confocal images of nes-jRGECO1a (left), *Per2-nls-Venus* (center), and merged images on the left side of the SCN slices. The center region of the left SCN is depicted. 3V: the third ventricle, OC: optic chiasma. (C) Representative traces of the Ca^2+^ and *Per2* rhythms. After recording Ca^2+^ and *Per2* rhythms for 4 days at 35°C, SCN slices were exposed to 15°C for 4 days, followed by rewarming to 35°C. All traces are average signals in the SCN region. (D) The plots of the peak phases of the Ca^2+^ rhythm (left), *Per2* rhythm (center), and phase difference (right). Time is depicted after the start of the recording. The data are presented as the mean ± SEM.

We expressed Venus under *Per2* promoter (*pAAV-Per2-intron2-Venus-NLS-D2*; *Per2-nls-Venus*) together with nes-jRGECO1a in the SCN slices (Figure 6A). Similar to in the case of *Bmal1*, we confirmed that *Per2-nls-Venus* was expressed exclusively in the nuclei of the SCN neurons (Figure 6B). We observed that the Ca^2+^ and *Per2* rhythms were stable with a phase difference (7.9 ± 1.3 h). Then, the SCN slices were cooled at 15°C for 4 days, followed by rewarming to 35°C for another 4 days (Figure 6C). We observed that as in the case of *Bmal1*, the Ca^2+^ and *Per2* rhythms were not detectable at 15°C, accompanied by a baseline level increase (181.1 ± 11.3%, 55.7 ± 9.0%, respectively). The Ca^2+^ increase was confirmed to be significantly larger than mApple in mouse SCN neurons (p = 0.0002). Notably, unlike in the case of *Bmal1*, both the Ca^2+^ and *Per2* rhythms were restarted quickly on Day 1 after rewarming (Figure 6D, left and center). The peak phases of the Ca^2+^ and *Per2* rhythms were detected at 13.9 ± 0.1 h and 4.8 ± 0.6 h after rewarming, respectively (n = 9). In particular, the phase differences between Ca^2+^ and *Per2* rhythms were large on Day 1 after rewarming (14.9 ± 0.5 h), but gradually recovered toward the original phase difference after a transient state for 4 days (9.2 ± 0.6 h) (Figure 6D, right).

**Figure 7:**
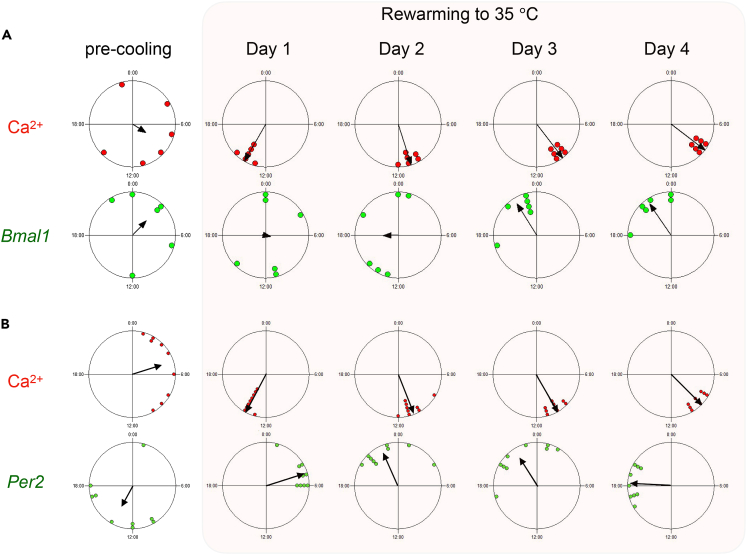
Rayleigh plots of Ca^2+^, *Bmal1*, and *Per2* rhythms before cooling and after rewarming (A and B) Rayleigh plots of the peak phase of the Ca^2+^ and *Bmal1* rhythms (A) and the Ca^2+^ and *Per2* rhythms (B). SCN slices were exposed under 15°C for 4 days, and the peak phases before cold exposure (pre-cooling) and after rewarming were calculated with respect to the start and end of cold exposure. The small circles on the Rayleigh circles indicate the phase of individual SCN slices, and the arrow lines within the circles indicate the average phase.

Figure 7 presents the Rayleigh plot analysis, showing that the Ca^2+^ rhythm restarted on Day 1 and the *Bmal1* rhythm gradually converged to a similar phase on Day 4, re-establishing the anti-phase relationship (Figure 7A). However, the *Per2* rhythm restarted on Day 1, with a larger phase difference relative to that of the Ca^2+^ rhythms. The Ca^2+^ and *Per2* phase differences were gradually re-established after a transient period of 4 days (Figure 7B). These results suggest that the Ca^2+^ rhythm differentially sets the phase of the *Bmal1* and *Per2* transcriptional rhythms.

### Cold-responsive Ca^2+^ rhythms in single SCN neurons

The SCN comprises subregions and multiple types of neurons. AVP- and VIP-expressing neurons are predominantly located on the dorsal and ventral subregions of the SCN, respectively.^1^ To assess SCN neuronal cold sensitivity at the single-cell level in different subregions, we sparsely expressed jGCaMP8s, a highly sensitive Ca^2+^ probe,^38^ in SCN slices using a mixture of two AAVs (hSyn-Flex-GCaMP8s and diluted hSyn-Cre). We observed the same phenomenon as that observed in the case of nes-jRGECO1a and Twitch2B in single SCN neurons in dorsal and ventral subregions. The rhythms got suspended at 15°C, the Ca^2+^ level increased, and the rhythm reappeared after rewarming to 35°C (Figures S8A and S8B). These results indicate that SCN neurons in distinct subregions exhibit identical responses to cold. Furthermore, the Ca^2+^ rhythm phase between dorsal and ventral SCN neurons was nearly identical immediately after rewarming, but gradually recovered after a few days (Figure S8C), indicating network-mediated topological specificity of Ca^2+^ rhythms.

Astrocytes in the SCN reportedly exhibit circadian rhythms, synchronizing the SCN network and regulating animal behavior.^39^^,^^40^ hSyn-nes-jRGECO1a expression is neuron-specific, whereas *Bmal1/Per2-nls-Venus* is supposedly expressed in both neurons and astrocytes. To assess the contribution of astrocytes to our analysis, we infected AAVs expressing mCherry under the control of an astrocyte-specific GFAP promoter with *Bmal1/Per2-nls-Venus* in the SCN slices. We observed that GFAP-mCherry was predominantly expressed in the third ventricle and at the edge of the slice, with very little signal observed within the SCN region (5.1 ± 0.5 cells/100 μm^2^, n = 16 slices) (Figure S9). The double-positivity percentage for Venus and mCherry labeling among the total Venus-positive cells was only 5–7% in the SCN region (*Bmal1-nls-Venus*: 5.4 ± 0.7%, n = 8 slices, *Per2-nls-Venus*: 8.2 ± 1.3%, n = 8 slices). Based on these results, we conclude that most fluorescence signals observed in our analysis originated from SCN neurons.

### Time required to rhythm resetting

We examined the time necessary to reset the Ca^2+^ rhythm after rewarming. The SCN slices were exposed to 15°C and the duration of cold exposure was systematically varied for 6–48 h. Figures S10 and S11 present the representative Ca^2+^ and *Bmal1* rhythm traces and phase plots, respectively. Short cold exposures (6–24 h) delayed the phase but did not reset the rhythms (Figures S10A–S10C; Figures S11A–S11C). Notably, the rewarming from a 48-h cold exposure reset the Ca^2+^ rhythms on Day 1. In contrast, the *Bmal1* rhythm phases were widespread on Day 1 (SD = 9.5 h), but they gradually converged into an identical phase on Day 4 (SD = 1.6 h) (Figures S10D and S11D) and re-established the anti-phase relationship relative to the Ca^2+^ rhythms (13.0 ± 1.2 h).

**Figure 8:**
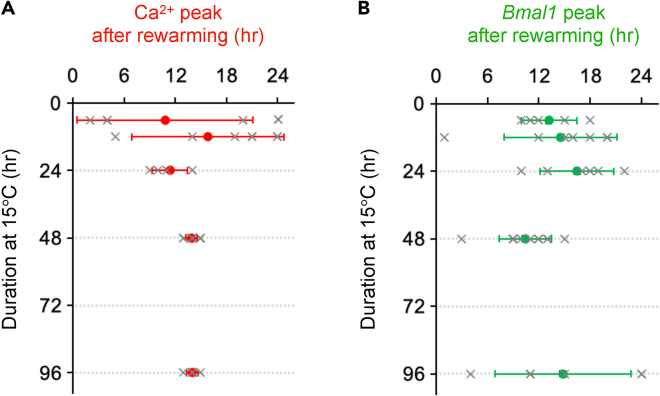
Relationship between cold exposure duration and peak phase after rewarming in SCN neurons (A and B) The 1^st^ peak phases of Ca^2+^ (A) and *Bmal1* (B) after rewarming. The duration of cold exposure at 15°C systematically varied from 6, 12, 24, 48, and 96 h. Individual data are represented by gray color x and mean ± SD by red/green colors.

Figure 8 summarizes the relationship between the duration of cold exposure and the 1^st^ peak phase of the rhythms after rewarming. After 6–24 h of cold exposure at 15°C, the Ca^2+^ rhythms were not reset (6, 12, and 24-h exposures: 10.8 ± 10.4, 15.9 ± 8.9, and 11.4 ± 2.1 h, respectively), but a 48-h cold exposure fully reset the Ca^2+^ rhythms (13.9 ± 0.7 h). In contrast, the *Bmal1* rhythms were not reset by any of the cold exposures (6, 12, 24, and 48-h exposures: 13.2 ± 3.3, 14.6 ± 6.6, 16.5 ± 4.3, and 10.4 ± 3.1 h, respectively). These results suggest that a cold exposure over 48 h would be required for the phase resetting of the Ca^2+^ rhythms.

### Circadian rhythms in Syrian hamster SCN

Finally, to validate the results obtained in mice in the case of hibernators, we performed key experiments in the SCN of Syrian hamster (*Mesocricetus auratus*) (Figure S12). Syrian hamsters enter a deep torpor that lasts for several days during winters and their core body temperature drops close to the ambient temperature of approximately 5°C.^41^ We infected AAVs encoding *Bmal1-nls-Venus* and nes-jRGECO1a in the SCN slices from hamster pups and confirmed the expression of these probes in the nucleus and cytoplasm of SCN neurons (Figures S12A–S12B). We observed *Bmal1* and Ca^2+^ rhythms (Ca^2+^: 24.3 ± 0.7 h, *Bmal1*: 22.4 ± 0.8 h) with a phase difference (8.6 ± 1.0 h) in hamster SCN at 35°C (n = 3 slices) (Figures S12C–S12E). Upon cold exposure at 15°C, both rhythms were suspended, accompanied by an increase in the Ca^2+^ level (72.2 ± 16.5%). Interestingly, the magnitude of Ca^2+^ increase in Syrian hamster SCN neurons was significantly smaller than that in mice (p = 0.0001), implying a species difference in cold response. After rewarming, the circadian rhythms resumed and returned to identical phases (Figure S12F). These observations in the SCN of Syrian hamsters and mice are essentially similar. To the best of our knowledge, these are the first live-cell recordings of circadian rhythms in hamster SCN.

## Discussion

Whether the clock gene transcriptional oscillations in the SCN neurons continue under hypothermia, specifically in the case of hibernation and daily torpor, has long been debated.^19^^,^^20^ A major reason for this question remaining unresolved is that brain samples were obtained from different animals to estimate temporal changes in clock genes and their protein expressions.^19^^,^^20^ This was based on the assumption that the circadian rhythm phase during hypothermia is identical in all animals. However, the accuracy of rhythm analysis significantly decreases if individual differences among animals are present, and the periods are not close to 24 h. To provide an insight into the clock gene transcriptional oscillations, it is crucial to evaluate circadian rhythms in identical living SCN tissue kept in a temperature-controlled environment. Moreover, a growing body of evidence supports the existence of a non-transcriptional oscillator without key clock genes,^42^^,^^43^ raising the possibility that the clock genes expression might not be an appropriate index for assessing circadian rhythms under hypothermic conditions. We recently demonstrated that Ca^2+^ signaling compensates for the slow transcriptional oscillation speed at low temperatures.^9^ Furthermore, we reported circadian Ca^2+^ rhythm persistence in SCN neurons lacking key clock genes *Cry 1/2*.^8^ These results suggest that not only are Ca^2+^ rhythms important for TTFL but they themselves are important for self-sustained, temperature-compensated oscillation. Therefore, we elucidated the mechanism of SCN rhythmicity mechanisms by simultaneously measuring clock genes and Ca^2+^ under various temperature conditions.

### Circadian rhythms under hypothermia

We discovered that the circadian rhythms of Ca^2+^, *Bmal1*, and *Per2* in the mouse SCN neurons were suspended at a cold temperature of 15°C. These results are consistent with those of previous reports using immunostaining or *in situ* hybridization in the brain samples of hibernating animals.^19^^,^^20^ During such deep hypothermia, clock gene and protein expressions were upregulated and suspended, and a neural activity marker was upregulated in the SCN.

This study primarily used the mouse SCN slice culture, which is an excellent model to study the cold response of circadian rhythms in mammals. However, the core body temperature of mice is not expected to drop to 15°C under physiological conditions. Mice undergo daily torpor that lasts only hours, not days, and their core body temperature do not drop below 20°C, suggesting that their SCN continues to function normally during daily torpor.

Interestingly, similar to the present results, the cyanobacterial circadian clock is lost at low temperatures around 19°C, and the self-sustained rhythm of cyanobacterial KaiC phosphorylation transformed to damped oscillations, as predicted by the Hopf bifurcation theory.^44^ Although the key molecules responsible for circadian oscillations differ between mammalian cells and cyanobacteria, the rhythm suspend at similarly low temperatures is intriguing from the aspect of clock function evolution.

Multiple Ca^2+^ sources reportedly contribute to Ca^2+^ rhythm generation in the SCN neurons, including tetrodotoxin-sensitive mechanisms via action potential firing and neuronal coupling, and Gq-Ca^2+^ signaling.^8^^,^^24^^,^^25^^,^^26^^,^^27^^,^^28^ Moreover, intracellular organelles such as the endoplasmic reticulum (ER) and mitochondria reportedly play an important role in Ca^2+^ rhythms generation.^24^^,^^45^ In skeletal muscles, cooling reportedly increases Ca^2+^ and tension,^46^ which is mediated by Ca^2+^ release from the internal stores. Although the origin of the cold-induced Ca^2+^ increase in SCN neurons remains unknown, Ca^2+^ release from ER might regulate the cold responses in the SCN.

Ca^2+^ signaling at low temperatures could be a key factor for rhythm suspension and resetting. Reported estimates of Ca^2+^ rhythm amplitude and baseline level vary depending on the Ca^2+^ probes and experimental conditions.^47^ Most estimates are based on Ca^2+^ probe properties (e.g., KD, Hill coefficient, dynamic range, etc.), which are measured *in vitro* at a given temperature. For example, the basal levels and peaks of Ca^2+^ rhythms using genetically encoded FRET probes are 119–440 nM,^24^ 85–120 nM,^26^ and 172–218 nM.^48^ In this study, the amplitude of Ca^2+^ rhythm and magnitude of the Ca^2+^ increase was approximately 30% and 200%, respectively, in SCN neurons at 15°C. Given the temperature sensitivity of mApple protein, the base fluorescent protein of jRGECO1a, the Ca^2+^ level would be 4.3 times the Ca^2+^ rhythm amplitude. For example, using a recent estimate in SCN neurons,^48^ estimated Ca^2+^ levels under cold at 15°C would be approximately 370 nM. Future studies should aim at accurate measurement of the absolute Ca^2+^ concentration achieved through further biochemical and physiological experiments.

### Theoretical insight: The hierarchy of Ca^2+^ oscillation and TTFL

In this study, we examined the time required to reset the circadian rhythms. Our results showed that 6–24 h of cold exposure were insufficient, but 48 h of exposure fully reset the Ca^2+^ rhythms in the SCN neurons. Upon rewarming, the *Bmal1* phase was widely distributed on Day 1, and the *Bmal1* rhythms gradually re-established the phase relation relative to the Ca^2+^ rhythms (Figure 6A). In comparison, the Ca^2+^ and *Per2* rhythms immediately restarted on Day 1. The *Per2* rhythm was phase-shifted gradually during the transient period and the original phase difference was restored relative to the Ca^2+^ rhythms (Figure 6B). At temperatures as cold as 22°C, the Ca^2+^ and *Bmal1* rhythms were transiently dissociated (Figure 4A), implying the presence of multiple oscillators in the SCN neurons.

These different properties of the Ca^2+^, *Bmal1*, and *Per2* rhythms could be explained by the limit cycle model, in which the amplitude of the limit cycle becomes smaller with decreasing temperature (Figure S13). The critical temperature for the rhythm suspension, a point of Hopf bifurcation, is 15°C–22°C. The transcriptional rhythm suspensions were accompanied by a significant Ca^2+^ increase, suggesting that Ca^2+^ signaling is a clock gene transcription rhythm regulator and a state variable of the limit cycles. *Per2* constitutes the core loop, which is regulated by Ca^2+^ via CaMKII and CRE.^1^^,^^3^ *Bmal1* is an essential element of the core loop regulated by RORs and REVERBs,^2^ but is not reported to be directly regulated by Ca^2+^. Interestingly, a recent study showed stable oscillations in cells and mice deficient for rhythmic transcription of the *Bmal1* gene by deletion of its upstream RRE elements.^49^ In addition, *Bmal1*-deficient SCN reportedly attenuates Ca^2+^ rhythms.^5^^,^^28^ Importantly, we described Ca^2+^ rhythms in *Cry1/2* deficient SCN neurons.^8^ These results indicate the hierarchy of Ca^2+^ and clock gene transcription as well as coupling directionality between the rhythms. The Ca^2+^ oscillator regulates *Per2*, which regulates *Bmal1*, *Bmal1* in turn regulates the Ca^2+^ oscillator.

Under cold temperature conditions of 15°C, the Ca^2+^ and *Per2* limit cycles transformed into damped oscillators and eventually suspend at a certain phase (Figures S13A–S13C), and restart from the identical phase after rewarming to 35°C (Figure S13C). In contrast, the *Bmal1* limit cycle damps slowly and suspends at various phases, and the phases after rewarming are variable and take several days to reestablish the phase relation with the Ca^2+^ rhythms. The phase difference between Ca^2+^ and *Per2* was initially beyond 12 h, but was gradually restored to its original value of ca. 7 h (Figure 6). These data suggest that the Ca^2+^ oscillator leads the phase of the oscillations of *Bmal1* and *Per2*.

### Limitations of the study

In this study, fluorescence time-lapse imaging on mouse SCN slices in a temperature-controlled environment allowed us to investigate in detail the characteristics of circadian rhythms at low temperatures. It has been reported that the physiological rhythms of hibernators are arrhythmic in the post-hibernation.^17^ The present study revealed unstable transcriptional rhythms in the SCN upon rewarming from cold temperatures. These results indicate that the unstable physiological rhythms of hibernators might be due to unstable SCN oscillations.

In this study, we used cultured SCN slices from neonatal animals as an experimental model and highlighted an important aspect of the SCN at cold temperatures. However, we cannot exclude the possibility that the adult SCN has a different cold response. Neonatal animals do not hibernate or enter torpor and are known to be resistant to hypothermia.^50^ In addition, neuronal circuitry and transmitter release in the SCN have been reported to undergo postnatal changes during development,^51^^,^^52^^,^^53^ and the changes in the periphery could alter the cold response properties that precede and coincide with hibernation.^54^ These postnatal and environmental factors may influence the cold responsiveness of the SCN. Thus, it will be crucial to elucidate the cold-responsive mechanisms in the adult SCN of hibernating animals in future studies.

The abdominal cavity and brain are considered to have similar temperatures during hypothermia,^55^ although a study described a slightly higher temperature in the brain than in peripheral tissue due to UCP-1 expression in mitochondria of the brain.^56^ This would require direct and continuous temperature measurements from the deep brain regions of freely moving hibernators. In addition, the cellular and molecular mechanisms by which rhythms are arrested at low temperatures and restart upon rewarming are unknown, and these would be important questions to be addressed in future research.

## STAR★Methods

### Key resources table

**Table:** 

REAGENT or RESOURCE	SOURCE	IDENTIFIER
**Bacterial and virus strains**		

AAV1-Syn1-nes-jRGECO1a	Addgene	100854-AAV1
AAV1-*syn*-FLEX-jGCaMP8s-WPRE	Addgene	162377-AAV1
AAV1.hSyn.Cre	Addgene	105553-AAV1
AAV1-hSyn1.Twitch2B	Addgene	100040-AAV1
AAV5-GFAP104-mCherry	Addgene	58909-AAV5

**Chemicals, peptides, and recombinant proteins**		

SYTOX-Green	ThermoFisher Scientific	S7020
Mineral Oil	Sigma–Aldrich	8042-47-5
α-tocopherol	Tokyo Chemical Industry Co., Ltd	T2309
ionomycin	ThermoFisher Scientific	I0634

**Critical commercial assays**		

Ni-NTA agarose	QIAGEN	Cat. No. 30210
PD-10 column	Cytiva	Code:17043501
illustra NAP-5 column	GE Healthcare	Code:17-0853-01
calcium calibration buffer kit #1	ThermoFisher	C3008MP

**Experimental models: Organisms/strains**		

Mouse: C57BL/6J	Japan SLC, Inc.	http://www.jslc.co.jp/english/
Syrian hamster: Slc:Syrian	Japan SLC, Inc.	http://www.jslc.co.jp/english/

**Recombinant DNA**		

AAV-P(Cry1)-forward-intron336-Venus-NLS-D2	Addgene	110054
AAV-P(Per2)-DIO-intron2-Venus-NLS-D2	Addgene	110057
AAV-hSyn-Venus	vectorbuilder	https://en.vectorbuilder.com
AAV-hSyn-mApple	vectorbuilder	https://en.vectorbuilder.com

**Software and algorithms**		

Prism GraphPad	GraphPad Software	https://www.graphpad.com
Excel	Microsoft	https://www.microsoft.com/en-us/microsoft-365/excel
Oriana	Kovach Computing Services	https://www.kovcomp.co.uk/oriana/

### Resource availability

#### Lead contact

Further information and requests for resources and reagents should be directed to and will be fulfilled by the lead contact, Ryosuke Enoki (enoki@nips.ac.jp).

#### Materials availability

This study did not generate new unique reagents.

#### Data and code availability

• Microscopy and spectroscopy data reported in this paper will be shared by the lead contact upon request.
• This paper does not report original code.
• Any additional information required to reanalyze the data reported in this paper is available from the lead contact upon request.

### Experimental model and study participant details

We obtained female mice (C57BL/6J) with newborn pups and pregnant Syrian hamsters from the animal breeder (Japan SLC, Inc.). The animals were housed in our animal quarters under controlled environmental conditions (temperature: 22°C ± 2°C, humidity: 40 ± 20%, 12-h light/12-h dark, with lights on from 0800 to 2000 h for mice and 0700–2100 for hamsters). The light intensity was adjusted to approximately 100–200 lx at the cage surface. The animals were fed commercial chow (Labo MR Standard; Nosan Corporation) and tap water. All animal care and experimental procedures were approved by the Institutional Animal Care and Use Committee of the National Institute of Natural Sciences and performed according to the National Institute for Physiological Sciences guidelines (Approved No.22A044 and No.23A080).

### Method details

#### SCN slice culture

The brains of neonatal mice and Syrian hamsters (5-to-6-day-old for mice, 3-to-4-days-old for hamsters, both male and female) were obtained in the middle of the light phase under hypothermic anesthesia, and rapidly dipped into an ice-cold balanced salt solution comprising 87 mM NaCl, 2.5 mM KCl, 7 mM MgCl_2_, 0.5 mM CaCl_2_, 1.25 mM NaH_2_PO_4_, 25 mM NaHCO_3_, 25 mM glucose, 10 mM HEPES, and 75 mM sucrose. A 200-μm coronal slice containing the mid-rostro-caudal region of the SCN was carefully prepared using a vibratome (VT 1200; Leica) and explanted onto a culture membrane (Millicell CM; pore size, 0.4 μm; Millipore) in a 35-mm Petri dish containing 1 mL of Dulbecco’s Modified Eagle Medium (DMEM) (Invitrogen) and 5% fetal bovine serum (Sigma–Aldrich). Prior to recording, the culture membrane was transferred onto a glass-bottom dish (35 mm, 3911-035; IWAKI) and the dishes were sealed with O_2_-permeable filters (membrane kit, High Sens; YSI) using silicone grease compounds (SH111; Dow Corning Toray). We applied 500 μL of Mineral Oil (Sigma–Aldrich) onto the surface of the DMEM to prevent evaporation and condensation during the long recording in the temperature-changing environment. We added 10 μM α-tocopherol (Tokyo Chemical Industry Co., Ltd.) to DMEM to prevent ferroptosis.^41^ To evaluate cell viability, 0.1 μM SYTOX-Green was added to DMEM (ThermoFisher Scientific). To increase the Ca^2+^ concentration, 10 μM ionomycin (Sigma–Aldrich) and 20 mM CaCl_2_ were added to the DMEM.

#### AAV-mediated gene transfer into the SCN slices

*pAAV-Bmal-forward-intron336-Venus-NLS-D2* was generated by swapping the *Cry1* promoter of *pAAV-P(Cry1)-forward-intron336-Venus-NLS-D2* (Addgene) with a *Bmal1* promoter.^57^ *pAAV-Per2-intron2-Venus-NLS-D2* was generated by deleting the *LoxP* cassettes of *pAAV-P(Per2)-DIO-intron2-Venus-NLS-D2* (Addgene). The plasmids of *pAAV-hSyn-Venus* and *pAAV-hSyn-mApple* were designed and created at vectorbuilder (vectorbuilder Inc). AAV vectors were packaged by using the AAV Helper Free Expression System (Cell Biolabs, Inc., San Diego, CA) as previously described.^58^ Briefly, the packaging plasmids (pAAV-RC1 and pHelper) and transfer plasmid were transfected into HEK293T cells using the calcium phosphate method. A crude cell extract was obtained from transfected cells, and AAV vector particles were purified by serial ultracentrifugation with cesium chloride. The purified particles were dialyzed with phosphate-buffered saline (PBS) containing 0.001% Pluronic F-68 (Sigma-Aldrich, St. Louis, MO), followed by concentration with an Amicon 10 K MWCO filter (Merck Millipore, Darmstadt, Germany). The copy number of the viral genome (vg) was determined by real-time quantitative PCR. AAV1-Syn1-nes-jRGECO1a, AAV1-*syn*-FLEX-jGCaMP8s-WPRE, AAV1.hSyn.Cre, AAV1-hSyn1.Twitch2B and AAV5-GFAP104-mCherry were purchased from Addgene. AAV aliquots (0.8 μL) were inoculated onto the surface of the SCN slices on Days 4–5 of culture. The infected slice was cultured for a further 9–12 days before imaging. To examine the cold response of rhythms at single-cell levels, we sparsely labeled SCN neurons using a mixture of AAV (hSyn-Flex-GCaMP8s and 1/1000 diluted hSyn-Cre). The titer of all AAV was over 1.0 × 10^13^ genome copies/mL.

#### Intracellular Ca^2+^ and clock gene expression imaging

Bioluminescence imaging using luciferase reporter is a generally used standard method to observe circadian rhythms.^59^ However, it is based on a biochemical reaction and metabolism, such as luciferin-luciferase enzymatic reaction and the hydrolysis of ATP, the activity of which is also considered to be temperature-dependent. Therefore, we used fluorescence reporters to assess Ca^2+^ and transcription rhythmicity. The low-temperature time-lapse imaging system was composed of an EM-CCD camera (Evolve; Photometrics), an inverted microscope (IX70; Olympus), dry objectives (20×, 0.75 NA, UPlanSApo; Olympus), a stage incubator (CU-109; Live Cell Instrument), an XYZ controller, a filter wheel (MAC6000, Ludl Electronic Products) and an LED driver (LEDD1B, Thorlabs). The microscopic system was placed in the low-temperature controller (CRH-212, ESPEC CORP). The upper part of the inverted microscope was removed to fit into the low-temperature controller. The microscopic system was controlled by the MetaMorph software (Molecular Devices).

The time-lapse wide-field imaging was conducted with an exposure time of 100 ms at 1-h intervals. Venus and jRGECO1a were excited by cyan (480/17 nm, Semrock) and yellow color (580/14 nm) with the LED light source (Spectra X Light Engine; Lumencor Inc) and the fluorescence was visualized with a dual-edge dichroic mirror (FF505/606-Di01, Semrock) and emission filters (FF01-530/43, FF02-641/75, Semrock). For FRET imaging using Twitch2B, mCerulean was excited by blue color (438/24 nm) with the LED light source, and mCerulean and cpVenus fluorescence was visualized with a dichroic mirror (FF458-Di02-25x36, Semrock) and emission filters (FF01-483/32-25, FF01-542/27-25, Semrock). We continuously perfused 5% CO_2_ with a gas mixer (GM-2000, Tokai-hit). Tissue and cell conditions were monitored by cultured slice bright-field images and calcium signals in individual neurons. The SCN slices were prepared from pups of the same mother and multiple imaging data were obtained in a single imaging experiment using a multi-dish holder and controllable XYZ-stages.^60^ The SCN slices prepared from pups of the same mother had similar phases of the rhythms. The whole SCN region and single SCN neurons were manually selected for the data analysis. ΔF/F_0_ = (F(t) - F_0_)/F_0_, where F(t) is the fluorescence value at a given time and F_0_ is the minimum fluorescence value. One round of cooling was performed per experiment.

#### Purified protein spectroscopy

nes-jRGECO1a was cloned into a pRSETb vector. Using this vector, *E. coli* (JM109(DE3)) was transformed and cultured in 200 mL of liquid LB medium at 23°C for 72 h with 140 rpm rotation. E. coli was collected by centrifugation, resuspended in PBS, and crushed using a sonicator. The recombinant protein was purified from the supernatant using Ni-NTA agarose (QIAGEN) and a PD-10 column (Cytiva), followed by exchange with TN buffer (50 mM Tris-HCl pH7.4, 300 mM NaCl) using the illustra NAP-5 column (GE Healthcare). Purified nes-jRGECO1a was characterized in 30 mM MOPS, 100 mM KCl, pH 7.2 (calcium calibration buffer kit #1, C3008MP, ThermoFisher) containing either 10 mM CaEGTA (calcium buffer) or 10 mM EGTA (EGTA buffer). Fluorescence spectra were measured with excitation at 400–600 nm (5 nm bandpass) and emission at 500–700 nm (5 nm bandpass) at temperatures of 5°C–35°C with 5°C increments using a fluorescence and absorbance spectrometer (Duetta, Horiba). The pH of the solutions at each temperature was measured using a pH meter (Horiba). The obtained data (excitation/emission wavelengths of 555 nm/600 nm) was plotted as the log of the [Ca^2+^]_free_ (x axis) versus the log {(F − F_min_)/(F_max_ − F)} (y axis) and the K_D_ and Hill coefficients were calculated from the linear fitting. The [Ca^2+^]_free_ was calculated from the K_d_ of EGTA for Ca^2+^ using the following equation.

$${\left[{Ca}^{2+}\right]}_{free}={K_{d}}^{EGTA}\times \frac{\left[CaEGTA\right]}{\left[K_{2}EGTA\right]}$$

Correction of K_d_^EGTA^ for pH was calculated by the following equation.^61^

$${K_{d}}^{EGTA}=\frac{\left[1+10^{\left(\log K_{1}-pH\right)}+10^{\left(\log K_{1}K_{2}-2pH\right)}\right]}{K_{Ca}}$$

Ponton association constant K_1_ and K_2_ are 10^9.58^ M^−1^ and 10^8.96^ M^−1^, respectively, and the primary Ca association constant K_Ca_ is 10^10.97^ M^−1^ at 20°C. The gas constant R is 1.9872 × 10^−3^ kcal·K^−1^mol^−1^ T, ΔH of K_Ca_ is −8.0 kcal mol^−1^, ΔH of K_1_ is −5.8 kcal mol^−1^, ΔH of K_2_ is −5.8 kcal mol^−1^, and T represents absolute temperature in kelvin. These values are put into the following Van’t Hoff isochore and the ponton and Ca association constants K′ for each temperature were calculated.^61^^,^^62^

$$\log\;K^{\prime }=\log\;K+\frac{\Delta H\left(1/T-{1/T}^{\prime }\right)}{2.303RT}$$

### Quantification and statistical analysis

Statistical analyses were performed using Excel (Microsoft) and Prism GraphPad (GraphPad Software). The imaging data were detrended by a 24-h running average subtraction method, and the peak phases of the rhythms were estimated by the peak phase of the rhythms. The first peak of *Bmal1/Per2* immediately after rewarming might reflect an acute response of the reporter proteins, we thus analyzed the second peak of the rhythms. The group mean was presented as the mean ± SEM. Circular plots were generated using Oriana (Kovach Computing Services). One-way ANOVA with a post-hoc Dunnett’s post-test was used to validate the temperature effects when paired multiple group data were compared within the same groups. Paired or unpaired t-tests were used when comparing two dependent and independent group means.
