# Supplementary material for: Cold-induced suspension and resetting of Ca2+ and transcriptional rhythms in the suprachiasmatic nucleus neurons
Source: iScience. 2023 Nov 3;26(12):108390. doi: 10.1016/j.isci.2023.108390 (PMC10700853; doi:10.1016/j.isci.2023.108390)
Supplement: Document S1. Figures S1–S13 [file mmc1.pdf]

iScience, Volume *26*

Supplemental information

Cold-induced suspension and resetting of Ca^{2+} and transcriptional rhythms in the suprachiasmatic nucleus neurons

Ryosuke Enoki, Naohiro Kon, Kimiko Shimizu, Kenta Kobayashi, Sota Hiro, Ching-Pu Chang, Tatsuto Nakane, Hirokazu Ishii, Joe Sakamoto, Yoshifumi Yamaguchi, and Tomomi Nemoto

Supplemental Information

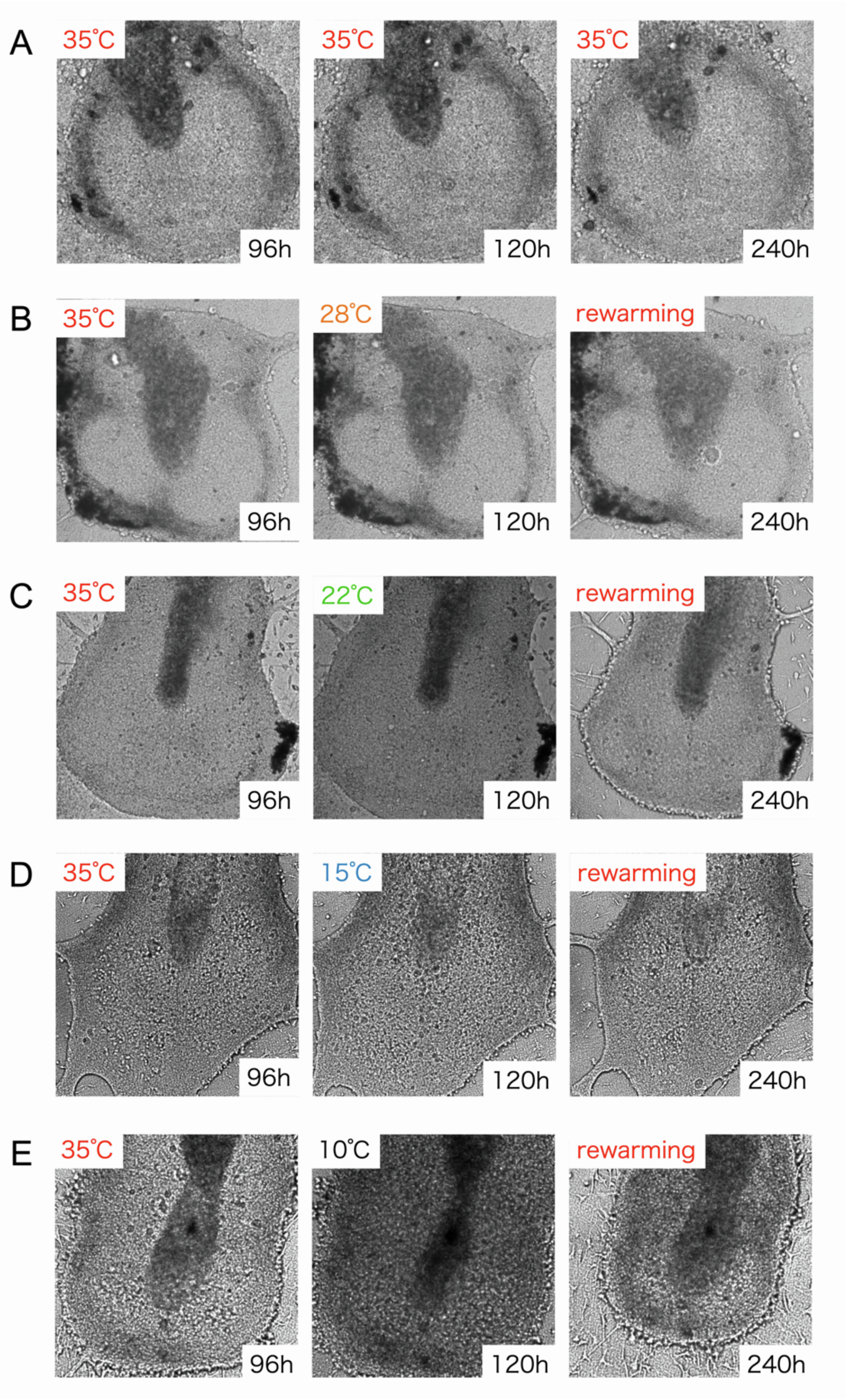

Figure S1. Cold-induced damage in mouse SCN slices. Related to Figure 2. Bright-field images before (left), during (center), and after (right) cold exposure. From top to bottom: 35°C, 28°C, 22°C, 15°C, and 10°C exposure, as indicated in the top left corner. The elapsed time after the start of recording is shown in the lower right corner.

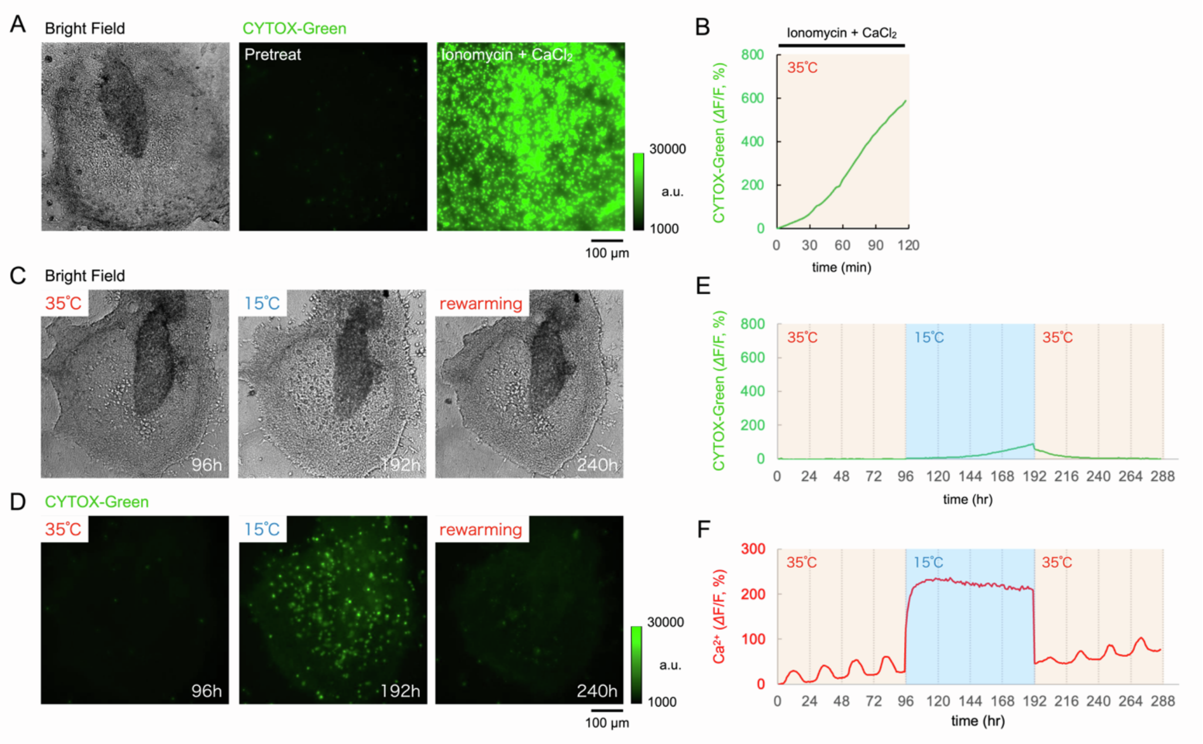

Figure S2. Evaluation of cell viability upon cold exposure. Related to Figure 2 and Figure S1. (A) Brightfield (left) and SYTOX-Green images of SCN slices before (middle) and after (right) ionomycin and $CaCl_2$ administration. (C-D) Brightfield and SYTOX-Green images of SCN slices before (left), during (middle), and after (right) cold exposure at 15°C. (E-F) SYTOX-Green and nes-jRGECO1a signal timecourses (corresponding to C and D).

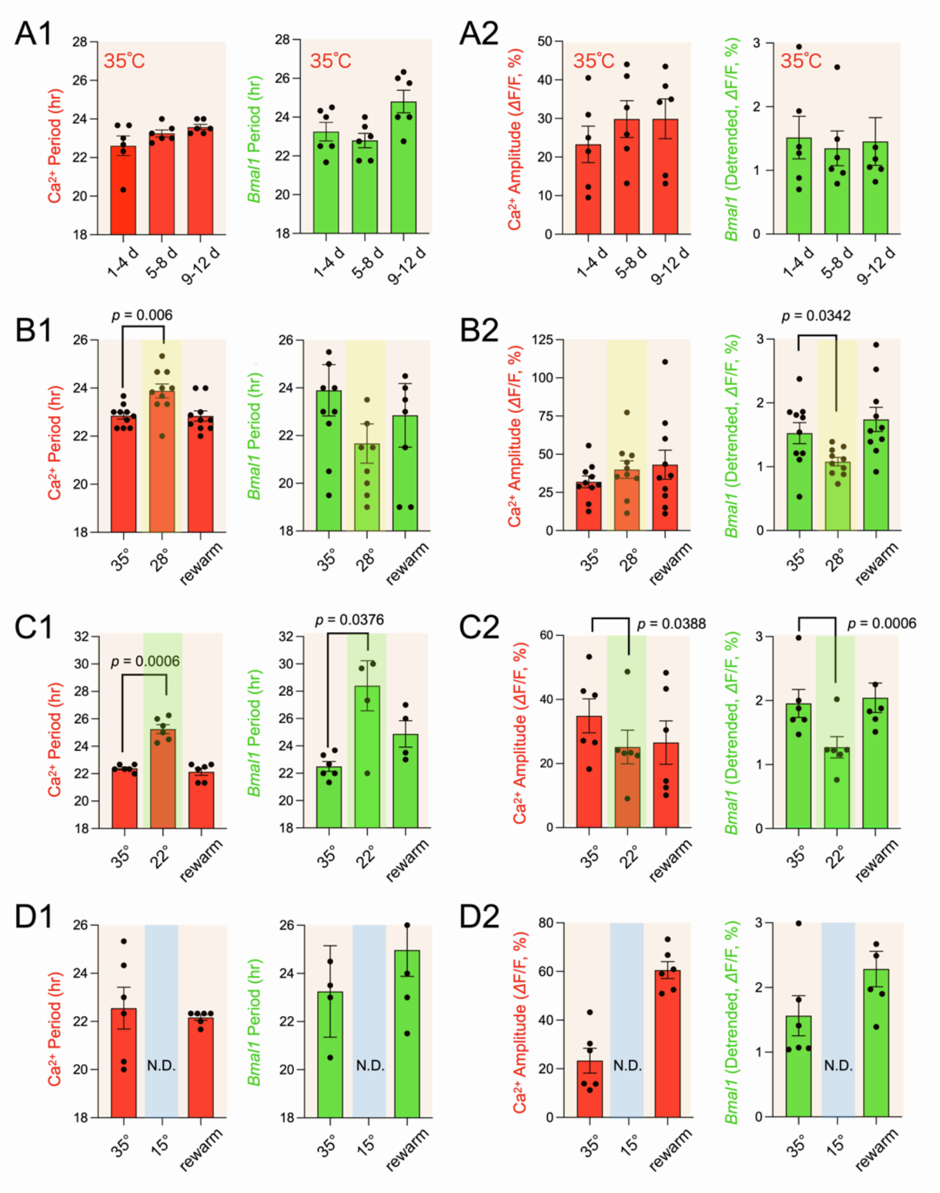

Figure S3. Statistical comparisons of Ca^{2+} and *Bmal1* rhythm. Related to Figure 2. Amplitudes (A1, B1, C1, and D1) and periods (A2, B2, C2, and D2) of Ca^{2+} (red, left) and *Bmal1* (green, right) rhythms. Graphs from top to bottom: 35°C (A), 28°C (B), 22°C (C), and 15°C (D). The data are presented as the mean ± SEM.

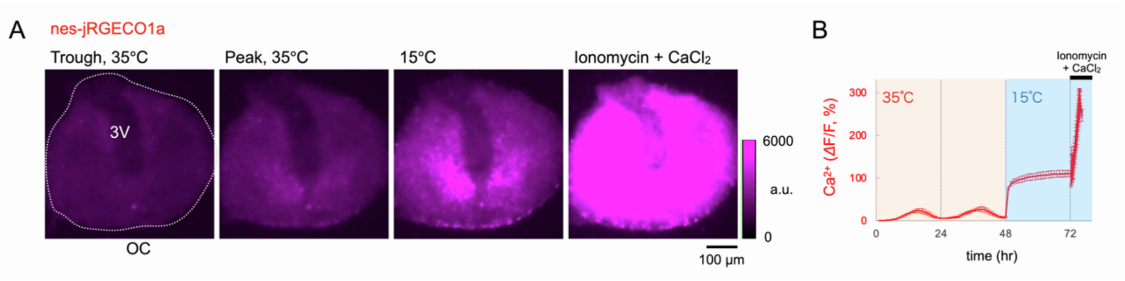

Figure S4. Evaluation of nes-jRGECO1a signals. Related to Figure 5. (A) Representative images of mouse SCN slice expressing nes-jRGECO1a under different conditions. Images from left to right show the trough and peak signals at 35°C, the maximum signal at 15°C, and the saturated signal caused by addition of ionomycin and $CaCl_2$. (B) Time course of nes-jRGECO1a signals. After recording signals at 35°C for 2 days, SCN slices were exposed to cold at 15°C for 1 day and then ionomycin and $CaCl_2$ were added. Time is depicted after the start of the recording. Data are presented as the mean ± SEM.

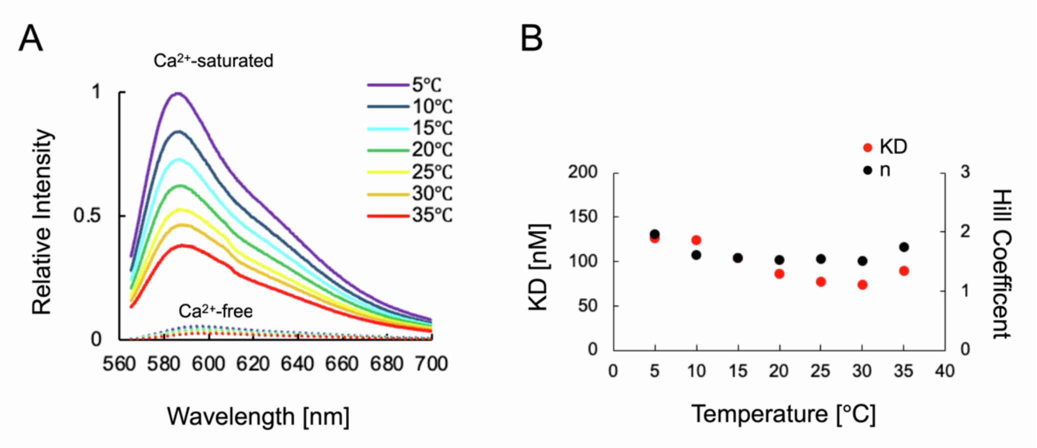

Figure S5. Biophysical properties of purified nes-jRGECO1a at various temperatures. Related to Figure 5 and Figure S4. (A) Emission spectra of nes-jRGECO1a in Ca^{2+}-free (dotted lines) and Ca^{2+}-saturated (solid lines) forms at 5-35°C. (B) The relationship between KD (nM) (red points) and Hill coefficient (n) (black points) of nes-jRGECO1a and temperature.

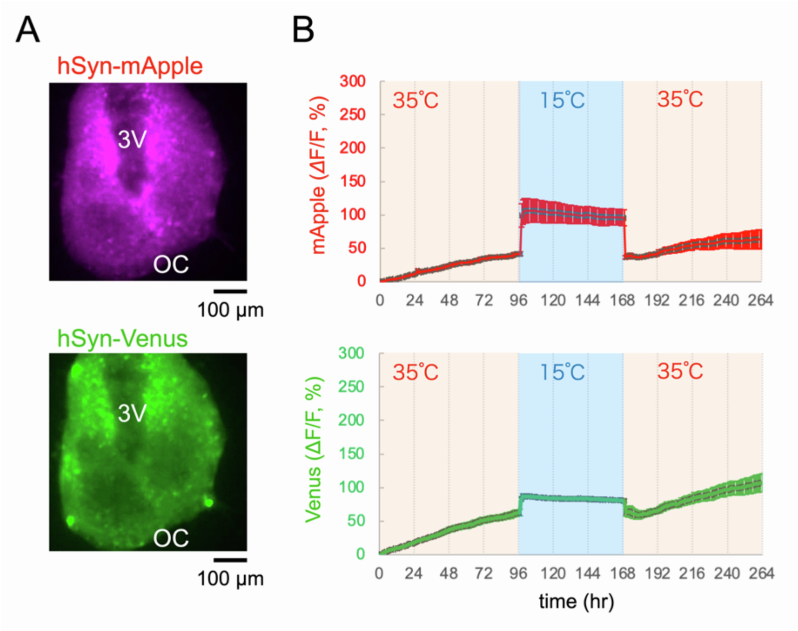

Figure S6. mApple and Venus fluorescence signals under cold exposure. Related to Figure 5. and Figure S5. (A) Representative images of SCN slices expressing mApple (top) and Venus (bottom). 3V: the third ventricle, OC: optic chiasma. (B) mApple and Venus signal timecourses. After recording signals for 4 days at 35°C, mouse SCN slices were exposed to cold at 15°C for 4 days followed by rewarming to 35°C. The traces represent mean signals of the entire SCN region. Time is depicted after the start of the recording. The data are represented as the mean ± SEM.

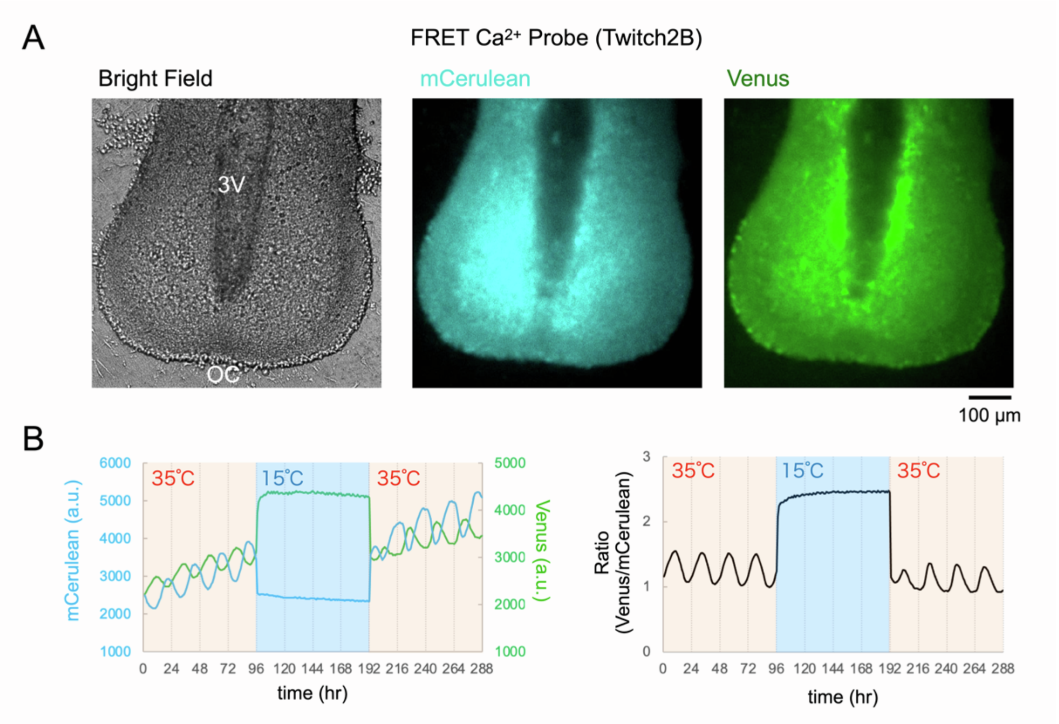

Figure S7. Circadian rhythms detected with FRET Ca^{2+} probes. Related to Figure 5. (A) Representative images of mouse SCN slices expressing the FRET Ca^{2+} probe Twitch2B. Brightfield (left), as well as fluorescent mCerulean (middle) and Venus (right) images. 3V: the third ventricle, OC: optic chiasma. (B) Representative traces of mCerulean and Venus (left), and the ratio (right). After recording signals for 4 days at 35°C, SCN slices were exposed to 15°C for 4 days, followed by rewarming to 35°C for 4 days. All traces represent average signals in the SCN region. Time is depicted after the start of the recording.

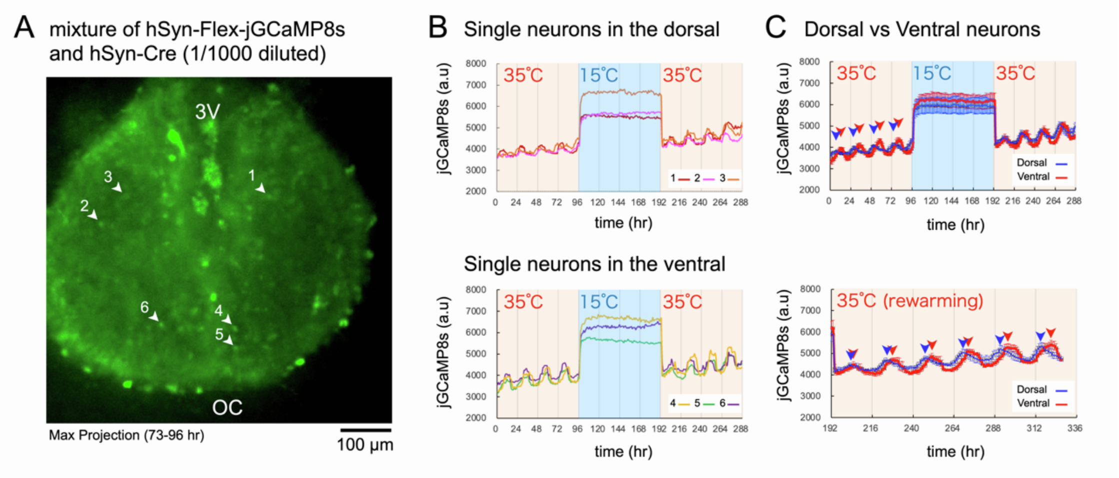

Figure S8. Single-cell analysis of cold-responsive Ca^{2+} rhythms. Related to Figure 5. (A) SCN slice sparsely expressing jGCaMP8s. A mixture of two AAVs, hSyn-Flex-GCaMP8s and diluted hSyn-Cre, was added to the SCN slices (see STAR Methods). (B) Ca^{2+} rhythms were detected in the dorsal (top) and ventral (bottom) SCN subregions. The position of the analyzed neurons is indicated by the arrowheads in (A). The Ca^{2+} rhythms were suspended at 15°C, Ca^{2+} levels increased, and the rhythm reappeared after rewarming to 35°C. (C) Mean Ca^{2+} rhythm traces of dorsal and ventral SCN neurons. The arrowheads indicate the rhythm peaks. Note that the peak phase of dorsal SCN neurons before cold exposure precedes that of ventral SCN neurons. Immediately after rewarming, the phase difference is small, but the phase relation is gradually recovered after several cycles.

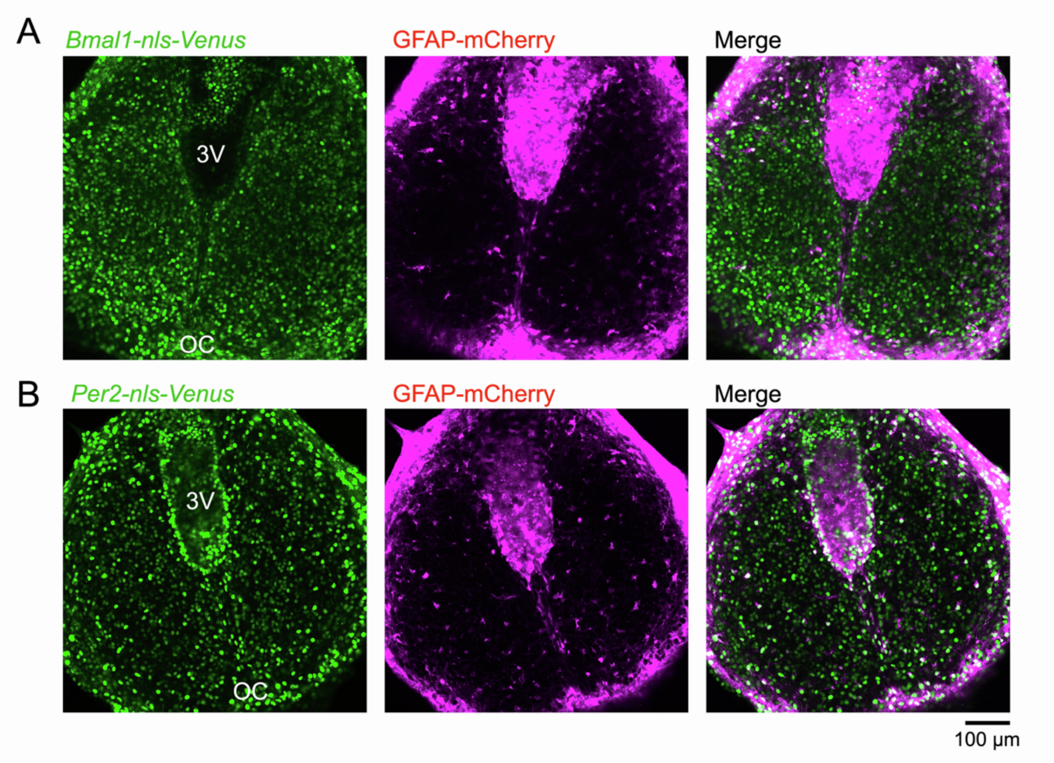

Figure S9. Expression patterns of glial cells. Related to Figure 1 and Figure 6. (A) Images of SCN slices expressing *Bmal1-nls-Venus* (left), GFAP-mCherry (middle), and the merged image (right). (B) Images of SCN slices expressing *Per2-nls-Venus* (left), GFAP-mCherry (middle), and the merged image (right). Note that cells expressing *Bmal1-nls-Venus* and *Per2-nls-Venus* are present throughout the SCN slice, whereas those expressing GFAP-mCherry are mainly present in the third ventricle and at the edge of the slice, with little expression within the SCN region.

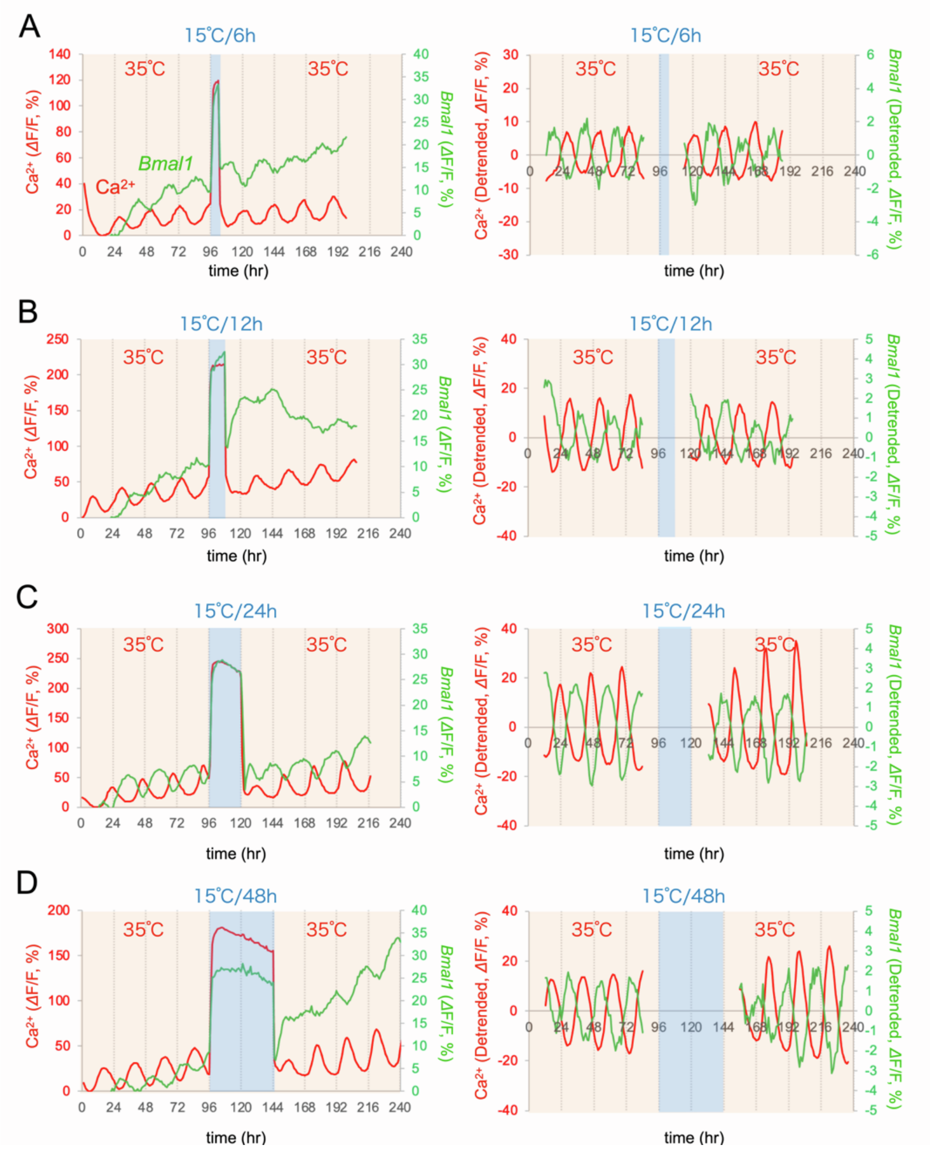

Figure S10. Representative traces of Ca^{2+} and *Bmal1* rhythms in SCN neurons with varying cold exposure times. Related to Figure 8.

Representative raw (left) and detrended (right) traces of ΔF/F (%). After recording Ca^{2+} and *Bmal1* rhythms for 4 days at 35°C, SCN slices were exposed in cold for 6 h (A), 12 h (B), 24 h (C), and 48 h (D) at 15°C, followed by rewarming to 35°C for 4 days. All traces are averages in the SCN region. Time is depicted after the start of the recording.

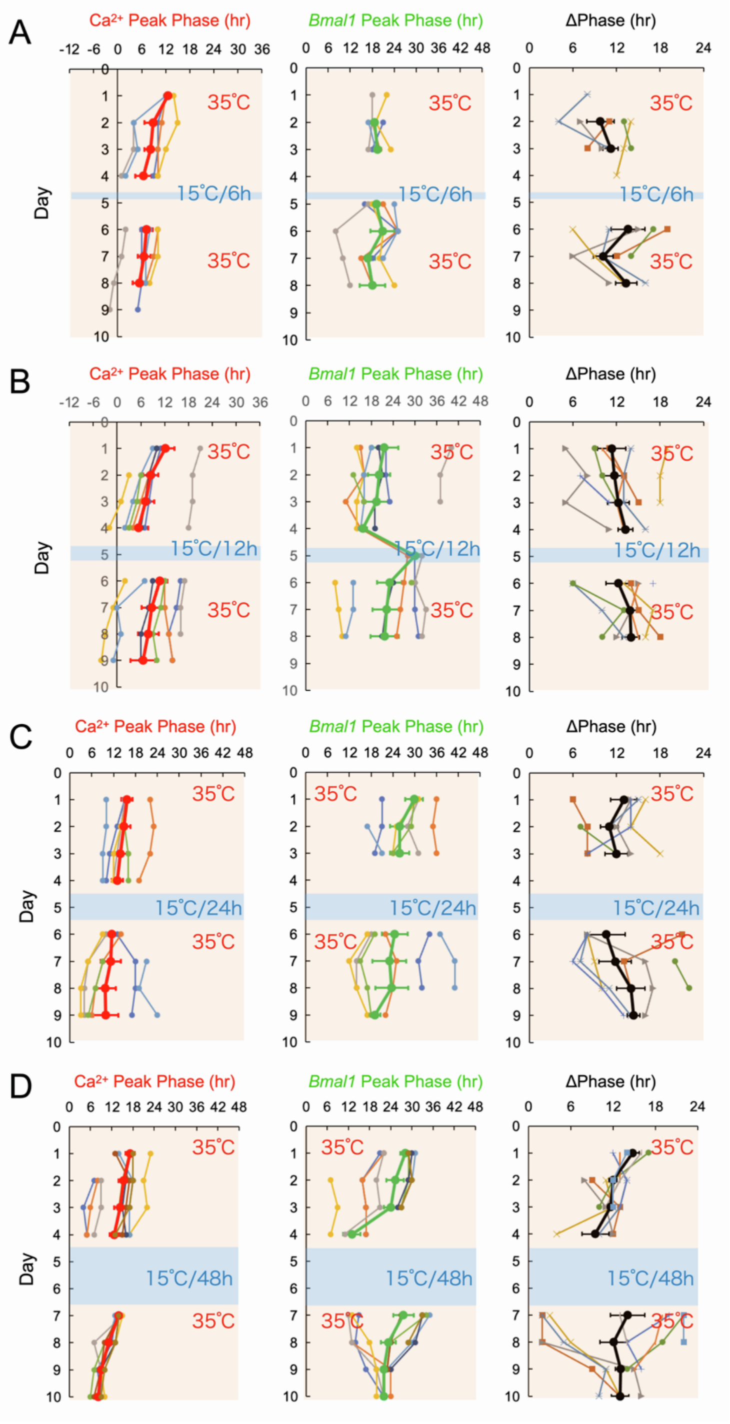

Figure S11. The plots of the peak phases of the Ca^{2+} and *Bmal1* rhythms and phase difference in SCN neurons with varying cold exposure times. Related to Figure 8. After recording Ca^{2+} and *Bmal1* rhythms for 4 days at 35°C, mouse SCN slices were cold-exposed for 6 h (A), 12 h (B), 24 h (C), and 48 h (D) at 15°C, followed by rewarming to 35°C for 4 days. All traces are averages in the SCN region. Individual slice data and average data are shown. The mean data are presented as the mean ± SEM. Time is depicted after the start of the recording.

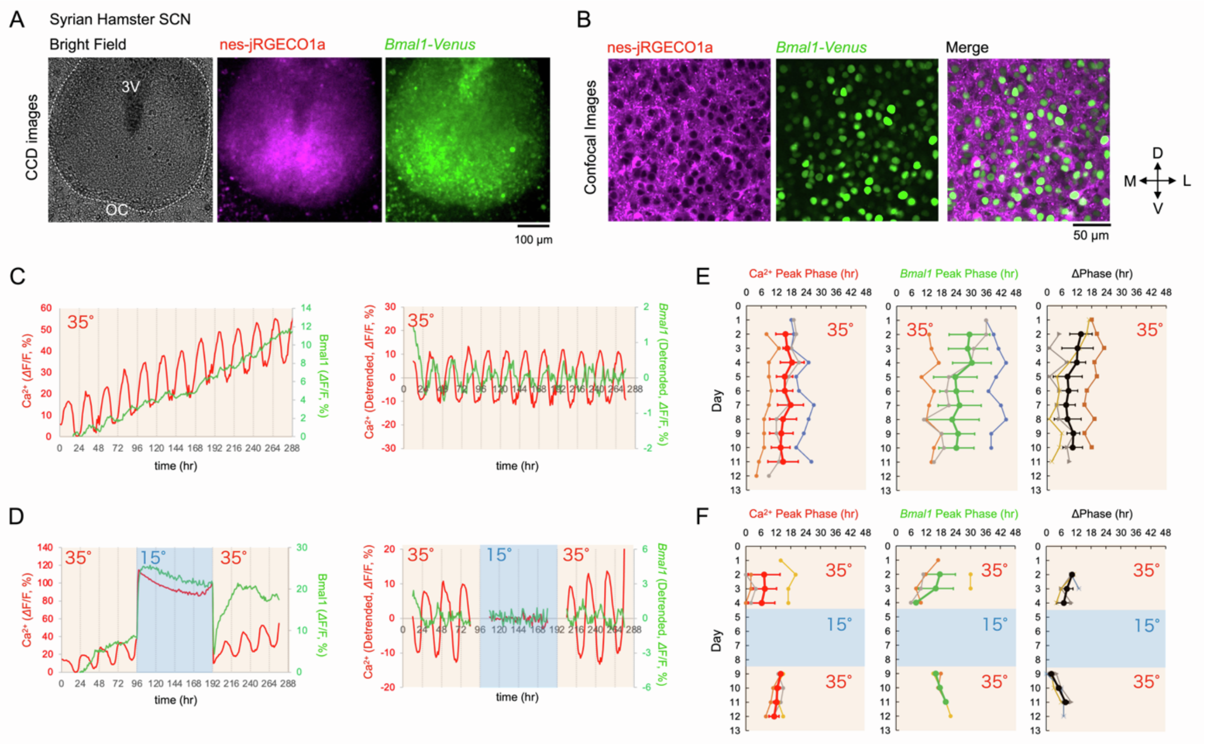

Figure S12. **Cold exposure suspends and resets the circadian rhythms in the SCN of Syrian hamsters. Related to Figure 1, Figure 2. Figure 3., Figure 5. and Figure 6.** (A) Representative images of Syrian hamster SCN slice expressing nes-jRGECO1a and *Bmal1-nls-Venus*. Brightfield (left), nes-jRGECO1a (center), and *Bmal1-nls-Venus* (right). (B) Confocal images of nes-jRGECO1a (left), *Bmal1-nls-Venus* (center), and merged images. (C) Representative traces of the Ca^{2+} and *Bmal1* rhythms maintained at 35°C. (D) After recording Ca^{2+} and *Bmal1* rhythms for 4 days at 35°C, SCN slices were exposed to 15°C for 4 days, followed by rewarming to 35°C. All traces in (C) and (D) are mean signals in the whole SCN region, and the *Δ*F/F (%) and the detrended traces are shown on the left and right, respectively. (E and F) The plots of the peak phases of the Ca^{2+} and *Bmal1* rhythm (left and center, respectively) as well as the phase difference (right) at 35°C (E) and upon cold exposure (F). Time is depicted after the start of the recording. The figure contains individual slice and average data. The mean data are represented as the mean ± SEM.

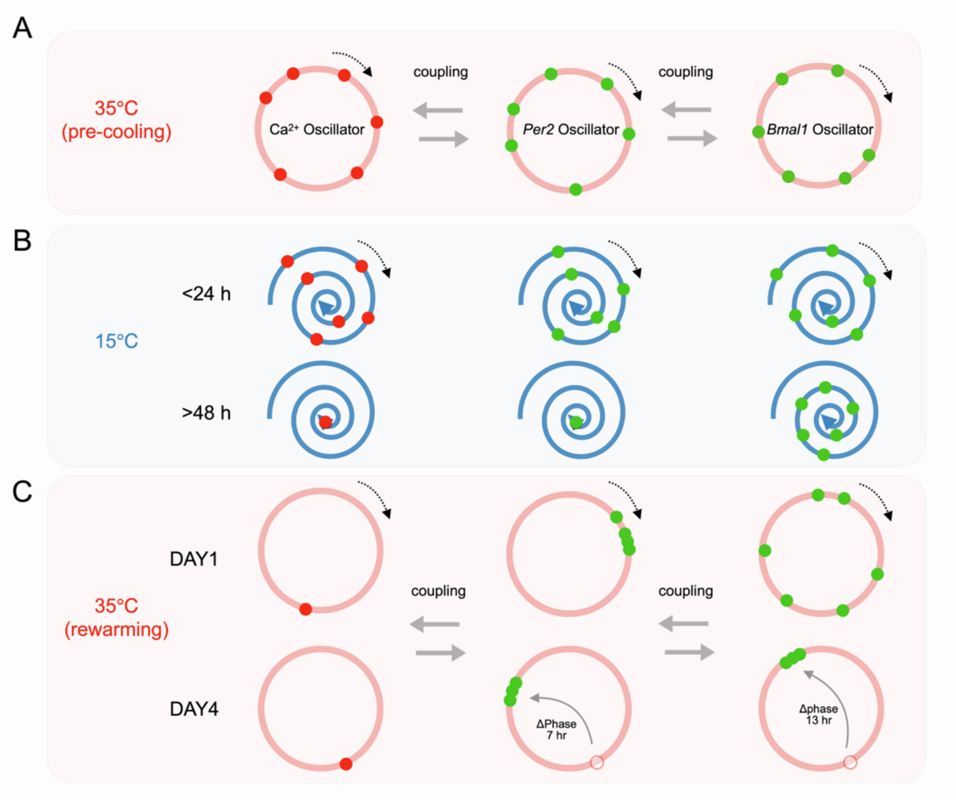

Figure S13. **Limit cycle model of cold-responsive circadian rhythms. Related to Figure 7 and Figure 8.** (A) The Ca^{2+}, *Per2,* and *Bmal1* oscillators are coupled at 35°C. The dots on the circles represent the circadian phase of the individual SCN slices. (B) Under severe cold at 15°C, the Ca^{2+} and *Per2* rhythms are transformed to the damped oscillator, which slow down and eventually get suspended at a certain point after 48 h cold exposure. The *Bmal1* limit cycle damps slowly and suspends at various phases. (C) Upon rewarming from the cold, the Ca^{2+} and *Per2* rhythms restart from the identical phase after rewarming to 35°C. In contrast, the *Bmal1* phases are variable on DAY1, taking several days to resynchronize to the Ca^{2+} rhythms with anti-phase relation (DAY4). *Bmal1* and *Per2* gradually restored the phase relationship relative to the Ca^{2+} rhythm during a transient period.
